# The timing of parental unemployment, insurance and children's education

**DOI:** 10.1080/14616696.2023.2188550

**Published:** 2023-03-22

**Authors:** Gabriele Mari, Renske Keizer, Ruben van Gaalen

**Affiliations:** a Erasmus University Rotterdam, Rotterdam, Netherlands; b Statistics Netherlands, Den Haag, Netherlands; c University of Amsterdam (UvA), Amsterdam, Netherlands

**Keywords:** Parental unemployment, educational outcomes, intergenerational effects, parental wealth, unemployment benefits

## Abstract

The timing of parental unemployment can impact children's educational transitions. Previous research has mostly examined transitions to higher education, proxying timing in relation to children's age and often focusing on selective populations. We study unemployment's intergenerational effects at multiple stages of the educational career, and define timing relative to important crossroads within and across school years for a broader population of children. Further, we build on suggestive patterns in prior studies and test if and how parental unemployment's effects vary depending on the availability, level, and combination of private insurance (parental wealth) and public insurance (unemployment benefits). We rely on Dutch administrative data on cohorts of students born between 1992 and 1998 and observed around the time of the Great Recession. With a negative-control design, we find that paternal unemployment in 6th grade decreases children's chances of enrolling in the general and academic secondary-school tracks, but only in families with lower wealth. Effects are moderate and partly flow from lower performance in a high-stakes test in 6th grade. These effects are reduced when households receive larger unemployment benefit amounts, particularly above median values. In addition, paternal unemployment in 6th grade has long-term negative effects on postsecondary enrolment for children with lower relative wealth. Differently, we do not find evidence of timing effects for spells of paternal unemployment occurring around high-school graduation, nor when examining the timing of maternal unemployment. These findings can inform remedial interventions aimed at mitigating the negative effects of disruptive events on children's education.

At the outset of the COVID-19 pandemic, unemployment was again front and centre in the policy agenda after last gaining salience during the Great Recession of the late 2000s. More generous benefits have often been provided to those experiencing unemployment, and job-protection schemes have sought to prevent job losses altogether. After all, unemployment's scarring effects, from lifetime income to mental health, are well-documented (for a review, Brand 2015). Following seminal studies in sociology (e.g. Elder 1974; McLoyd et al. 1994), the negative impact of parental unemployment on children's educational achievement has also been established (e.g. Rege et al. 2011; Kalil and Wightman 2011; Gregg et al. 2012; Brand and Simon Thomas 2014; Lindemann and Gangl 2020). In particular, recent research has shed light on how not just the occurrence of parental unemployment but its timing relative to children's educational career, i.e. whether children are at a crossroads such as high-school graduation, can make a difference (e.g. Coelli 2011; Pan and Ost 2014). Yet, policies such as unemployment benefits do not account for the stage of formal education children are navigating at the time of parental unemployment. Lacking insurance from public provision, families' own financial resources can provide ‘private’ insurance. If private insurance mechanisms buffer risks for well-off children whilst exacerbating risks for the least well-off, costly socioeconomic gaps in education might be reinforced.

We study how the timing of parental unemployment might affect children's education, focusing on which families are better off depending on possible insurance mechanisms. We analyse Dutch register data for cohorts exposed to parental unemployment around the Great Recession. We propose, first, that parental unemployment may alter children's educational trajectories at multiple crossroads. We add to previous studies on enrolment in higher education, and, in addition, we examine parental unemployment occurring around the time of sorting into academic and vocational tracks in secondary school. Such sorting, common in various forms in Europe, can have adverse effects on the educational outcomes of students from disadvantaged socioeconomic backgrounds (e.g. Hanushek and Wößmann 2006; Van de Werfhorst 2019). Expanding current knowledge, we ask if parental unemployment might lead students to sort in vocational rather than general/academic secondary-school tracks. This question is particularly relevant in the Netherlands (and similar institutional contexts), as a high-stakes test in conjunction with early tracking at the end of primary school has already been shown to deepen socioeconomic disparities (Borghans et al. 2020; Zwier et al. 2020).

Our second contribution concerns the timing of parental unemployment and how and for whom research could gauge its effects. Studies have often differentiated unemployment based on children's age at the time of the spell (e.g. Coelli 2011; Pan and Ost 2014; Lehti et al. 2019). We complement this perspective by looking at educational crossroads *within* the school year in specific grades and capture such timing by matching children's educational trajectories to quarterly data on parental unemployment spells. As ‘for whom’, research has often restricted its analyses to unemployed parents following involuntary job loss (e.g. Rege et al. 2011) or comparing unemployment spells within families with at least two children in sibling designs (e.g. Lehti et al. 2019). Whilst these choices may address confounding bias, the composition and mechanisms at work in these samples might be distinctive (e.g. due to sorting across firms, spillovers across siblings, etc.). As a result, effects may fail to generalise to the larger pool of children with an unemployed parent. To maximise external validity, we retain all beneficiaries of unemployment benefits regardless of family size. We also focus on a period comprising a recession, when unemployment might be more diffused and the unemployed less selective than during times of growth. As for internal validity, we tackle confounding bias by comparing children exposed to unemployment in different quarters of the same year, in grades with ‘educational crossroads’ and without, using a negative-control design adopted from epidemiology (Lipsitch et al. 2010). In short, we add to prior literature by asking if and when timing matters within the school year and across grades among a broader population of children of unemployed parents than in most previous studies.

Third and last, we examine the role of private and public insurance mechanisms. Following sociological literature on wealth inequalities (Pfeffer 2011; Killewald et al. 2017; Hällsten and Thaning 2021), we posit that parental wealth provides private insurance to well-off families, a buffer to the stress and income loss via which parental unemployment might stray, if badly timed, children's educational path. For less well-off families, public provisions such as unemployment benefits might provide that timely buffer (e.g. Lindemann and Gangl 2020). Studies have either examined parental wealth in single-country studies (as proxied by homeownership and only in the US, Kalil and Wightman 2011; Pan and Ost 2014) or public policies in a comparative perspective (Lindemann and Gangl 2020). In a country where wealth disparities are wide (Balestra and Tonkin 2018) and unemployment insurance is relatively generous (Kalwij et al. 2018; de Groot and van der Klaauw 2019; De Nardi et al. 2021), we highlight instead the interaction between private and public insurance (Pfeffer and Hällsten 2012). We divide families based on direct measures of parental wealth–primarily, net worth–and look at how families across the wealth gradient fare depending on their unemployment benefit entitlements. We thus aim to unveil the stratification of unemployment's intergenerational effects. In doing so, our study also speaks to debates on the design and targets of remedial policies.

## 1. Background and previous research

### 1.1. Parental unemployment, children's education, and ‘bad’ timing

Our study aims and design are closely related to previous evidence on the timing of parental unemployment and children's education. Coelli (2011) found that parental job loss around high-school graduation lowered chances of enrolment at university and community college among Canadian youth. For the US, parental layoff just before graduation, rather than soon after, was found to decrease chances of college enrolment (Pan and Ost 2014). In an institutional context more similar to ours (Austria), university enrolment chances were also found to decrease following a spell of parental unemployment occurring just before tracking across secondary schools at around age ten (Schmidpeter 2020).

Evidence of such ‘bad’ timing could be motivated by two mechanisms, stress and investment. It is well-known that stress comes with job loss and unemployment, first for those experiencing unemployment themselves (for a review, Brand 2015) and then spilling over to their partners (e.g. Marcus 2013; Mendolia 2014). Further spillovers on children can be expected (for a review, Kalil 2013). In particular, if unemployment strikes around a (high-stakes) test or exam, it may jeopardise children's educational performance.

Little direct evidence supports this explanation, but several studies lend credence to such a ‘stress-around-the-test’ argument. First, recent changes in household socioeconomic circumstances, due to intra-year income instability (Gennetian et al. 2015) or dwindling support from social programs (e.g. for the Supplemental Nutrition Assistance Program [SNAP] in the US, Gassman-Pines and Bellows 2018), have been found to decrease children's test performance and school engagement. Research has also highlighted the biological processes via which stress may result in lower cognitive performance – for example, by affecting sleep and cortisol production (Heissel et al. 2017, 2021)–possibly contributing to test score differentials along the socioeconomic divide. A recent multi-country study has further supported the idea that high-stakes tests can lead to heightened self-reported stress (Högberg and Horn 2022). Last, whilst not zooming in on the timing of parental unemployment relative to tests or exams, the occurrence of parental job loss and unemployment *per se* has long been associated with lower educational performance among children, from grade-point averages to exam scores (Rege et al. 2011; Gregg et al. 2012; Lehti et al. 2019; Mörk et al. 2020).

Other than stress affecting school performance, unemployment may alter investments in education. Income losses following unemployment might offset parents' ability to finance tuition and keep up with living costs if children were to enrol in postsecondary education. With few exceptions (Coelli 2011; Pan and Ost 2014), studies have found little evidence for credit constraints when examining educational investments in college or university in households hit by unemployment (e.g. Hilger 2016). Around earlier educational transitions, though, and especially those dependent on school performance, unemployed parents might find it difficult to pay for so-called ‘shadow education’, those remedial and enriching educational activities (tutoring, extra classes, test preparation) that occur outside of the formal school day (Buchmann et al. 2010; Zwier et al. 2020). Alternatively, parental (and children's) investments in education may flow from a changed outlook to the future in the aftermath of unemployment. The latter might increase short-term uncertainty around parental income inflows. Around high-school graduation, affected children might discount the long-term returns of further investments in education as opposed to early entry into the labour market – to contribute more swiftly to household finances (see, e.g. Fradkin et al. 2019).

In sum, a badly timed spell of parental unemployment might precede not just enrolment in postsecondary education, but also earlier transitions in children's educational trajectory, especially when tied to a high-stakes test. Whilst performance in the latter might be affected by stress and investments in shadow education, the large financial commitment that comes with higher education might be hard to sustain in families hit by unemployment, either due to material costs or to parental and children's own assessments of opportunities in education as opposed to the labour market.

### 1.2. The interplay of private and public insurance: parental wealth and state transfers

Insurance mechanisms may moderate the intergenerational effects of parental unemployment. We focus on the kind of private insurance offered by parental wealth (e.g. Pfeffer 2011) and assess its interplay with public provisions that may absolve the same function, namely unemployment benefits (e.g. Lindemann and Gangl 2020).

A gradient in children's educational chances and achievement depending on parental wealth is well-documented (e.g. Pfeffer 2011, 2018; Hällsten and Thaning 2021; for a review, Killewald et al. 2017). Parental wealth may spur investments in education throughout children's life course -- via intergenerational transfers, networks, the choice of neighbourhood, etc. - but it may also provide ‘real and psychological safety nets’ (Shapiro 2004; Pfeffer 2011) in times of adversity. Even if experiencing unemployment, wealthier families may benefit from ‘real’ insurance consisting in the ability to repay any student loan or to finance investments in education via savings or home-equity lending (Kalil and Wightman 2011; Lovenheim 2011). Suggestive of such mechanisms, for example, a spell of parental unemployment around high-school graduation was found to hurt chances of postsecondary education enrolment among renters but not homeowners in the US (Pan and Ost 2014). On top of assets which can be liquidated or against which families can borrow, parental wealth may also offer ‘psychological’ insurance, mitigating stress and pessimistic outlooks to the future. Research has found that wealth indeed moderates associations between unemployment, on the one hand, and mental health or subjective well-being, on the other, at least among older adults (Riumallo-Herl et al. 2014; for the Netherlands, see also Müller et al. 2021). If such buffering effects also hold among (younger) parents and children, wealth may help defuse the ‘stress-around-the-test’ effects of parental unemployment.

The scope of wealth's private insurance function, nonetheless, may depend on how well public provisions can insure unemployed parents and their children (Pfeffer and Hällsten 2012; see also, e.g. DiPrete 2002; Gangl 2006; Sjöberg 2010). Studies have often pointed to the generosity of state-benefit programs in Nordic countries to account for the null or small intergenerational effects of unemployment observed in such contexts (Bratberg et al. 2008; Lehti et al. 2019; Mörk et al. 2020). Few have explicitly tested such a proposition or examined whether the least well-off families have more to benefit from public insurance. The exceptions have highlighted, for example, the role of unemployment benefits. Across Europe and the US, Lindemann and Gangl (2020) have found smaller associations between parental unemployment and entry into postsecondary education in contexts with more generous unemployment benefits. This moderating role of benefit generosity was somewhat weaker for children of college-educated parents, who ‘have more savings and other assets to protect themselves against unfavourable circumstances’ (*ibidem*, 621).

Both theory and evidence hence suggest that a spell of parental unemployment, even if badly timed, might have fewer consequences for children's education in wealthier families. Recently unemployed parents who cannot fall back on their own wealth, instead, might not be able to afford large expenses, such as those for university education, or might feel more stress, which may in turn impact children's school performance. In such families, unemployment benefits might provide insurance, although direct tests are few and far between.

## 2. The Dutch context

### 2.1. Crossroads in the Dutch educational system

An examination of timing and insurance effects should acknowledge that parental unemployment might interfere with children's educational trajectories at multiple crossroads in a given educational system. In the Netherlands (see, e.g. Scheerens et al. 2012 and Figure A1 in the Appendix), a first crucial crossroads happens at around age 12. After six years of primary school (6th grade or *Groep 8*^1^ in Dutch), students enter one of three main tracks for their secondary education: VMBO (vocational), HAVO (general) and VWO (pre-university). Only HAVO and VWO provide direct access to higher education, and thus tracking is consequential for children's future educational attainment and life chances more broadly.

In the period we consider, the sorting of students across tracks depended on two assessments. The first is a high-stakes test (in most schools, the so-called CITO test), evaluating students' language and maths skills, among others. Around two thirds of schools administered CITO tests, typically in the first week of February (except for 2008, when the test was held in the second week of February, and 2013, when the test was administered in April). The test is constructed to have a grand mean of 535 and a standard deviation of 10, and test developers provide standard thresholds to assign each student to a track based on their score. In the study period, the following thresholds were applied: VMBO-B (*Basisberoepsgerichte*) for scores between 501 and 523, VMBO-K (*Kaderberoepsgerichte*) from 524 to 528, VMBO-GT from 529 to 536 (*Gemengde*/*Theoretische*), HAVO from 537 to 545, VWO from 545 to 550.

After test results are known or around the same time of the academic year in schools not administering the CITO, 6th-grade teachers make their assessment. Teachers recommend one of the tracks for each student based on prior school performance, including the indication provided by the final test for those who took it. In most cases,^2^ the teacher recommendation and test score align, and the advice is *de facto* binding. Henceforth, we will refer to this crossroads occurring in 6th grade as the *school advice*.

A second crossroads is the transition from secondary to postsecondary education. After five years of general education (HAVO), students can attend higher professional education at so-called HBO. After six years of pre-university education in the academic track (VWO), students can enrol in university. Students from the HAVO track can attend university only if they switch to VWO and obtain a diploma there, or once they graduate from HBO. In the last year of secondary school, students apply for entry into postsecondary education. To graduate, students also take an exit exam around May of the same year, but sorting into HBO or university is dictated by their current track and final diploma and not by the exam results. Postsecondary education in the Netherlands is by and large public and non-selective, with tuition fees that are, on average, on par with neighbouring European countries but lower than the UK or Ireland (e.g. European Education and Culture Executive Agency [Eurydice] 2015).

### 2.2. Private and public insurance in the Netherlands

Context also matters to more fully grasp the role of private and public insurance. The Netherlands has one of the highest levels of wealth inequality among countries in the OECD group. According to estimates based on the OECD Wealth Distribution Database (Balestra and Tonkin 2018), for example, the estimated mean to median wealth ratio is over 8, only comparable to that of the US (whilst the same ratio in half of OECD countries stops at around 2). Wealth concentration in the top 1, 5, and 10% is the largest in Europe. When considering housing wealth alone, however, the Netherlands often figures among the most equitable contexts across high-income countries (Dewilde and Flynn 2021). At the bottom of the wealth distribution, households often muster considerable assets, counterbalanced though by their mortgages (Balestra and Tonkin 2018). This is particularly relevant in our study, for property values plummeted during the housing bust that followed the boom at the root of the Great Recession (for the Netherlands, Steegmans and Hassink 2017). Households with large mortgage debt, over and beyond their assets, might have faced significant uncertainties in the period, possibly worsened when adding unemployment to the equation. We will return to these pointers when discussing our findings.

As for public insurance, a new unemployment benefit scheme became effective in the Netherlands in October 2006 (Kalwij et al. 2018). The new scheme insures all employees against unemployment by offering a 3-month minimum period of benefit entitlement (see also de Groot and van der Klaauw 2019). Benefit amounts are tied to pre-unemployment wages, replacing 75% of the previous (daily) wage for the first two months of entitlement and 70% from the third month onwards. In a comparative perspective, although payments have become less generous and eligibility stricter over recent decades (Mooi-Reci and Mills 2012), public provisions in the Netherlands provide fairly extensive protections against job loss and unemployment (De Nardi et al. 2021).

## 3. Empirical approach

### 3.1. Data

We use register data from Statistics Netherlands (Bakker et al. 2014; replication files are available at https://osf.io/bc735/). Data comprise cohorts of children born between 1992 and 1998. We selected children in the 1992–1998 cohorts for they entered secondary and postsecondary education in a period that included the Great Recession. The recession provides us with an ideal setting for our questions. Whilst not as much as in other European countries or the US, unemployment rates increased in the Netherlands from 2009 onwards, peaking at around 8% for both men and women in 2013 and 2014 (OECD database). Our analyses start from 2006 due to data availability, as 2006 is the first year with available records on parental wealth at Statistics Netherlands.

We analyse two main educational outcomes. In line with previous studies (e.g. Coelli 2011; Pan and Ost 2014; Lindemann and Gangl 2020), we focus on children's educational enrolment chances. First, we examine a binary outcome contrasting children enrolled in the tracks that give access to higher education (HAVO or VWO) or not (VMBO). We measure this first outcome in the third year of secondary school, as students in the previous two years may still be enrolled in so-called bridge classes with a ‘mixed’ track curriculum, e.g. VMBO *and* HAVO (e.g. Borghans et al. 2020). Children in the third year of secondary school are around 15 years old, and thus our last available time point for this outcome is 2013. Second, we measure enrolment in higher education by age 20 with a binary indicator distinguishing those enrolled or not in any postsecondary education, be it HBO or university. The 1998 cohort is the last one for which we can measure this outcome (as students reach their 20s in 2018, the last record available to us for this second outcome).

We match children's educational records with quarterly information on parental unemployment spells and yearly data on parental wealth. The next paragraph provides details on which quarters and grades were selected and why. Cell sizes in each grade-by-year combinations are displayed in Figure A2 in the Appendix. As for unemployment, we consider *moves into* unemployment from any other state in the prior quarter. Unemployment is defined based on (successful) unemployment benefit claims, as registered in Dutch social security data. The recorded start of each spell corresponds to the day after the end of a person's last job. In line with past research (e.g. Rege et al. 2011), we conduct separate analyses for children exposed to paternal and maternal unemployment, as the latter has been found to have less clear-cut effects on children's education (for a review, Mörk et al. 2020).

We thus have four separate populations depending on whether we are analysing paternal (*p*) or maternal (*m*) unemployment and sorting in secondary school (*s*) or enrolment in postsecondary (*post*) education (Np,s=44,637 Nm,s=43,851; Np,post=38,328; Nm,post=38,216). Table 1 provides descriptive statistics. The study population is restricted to children who lived with married or cohabiting parents or with a single parent in the first quarter of the calendar year in question. When studying postsecondary enrolment, we also restrict our population to those enrolled in HAVO or VWO, i.e. those ‘at risk’ of enrolling into postsecondary education. This population of children is relatively more advantaged, as evidenced in Table 1, for example, by the large(r) share of children with at least one tertiary-educated parent. Further, we follow long-held practices in the study of unemployment and its consequences (e.g. Jacobson et al. 1993) and exclude records belonging to parents with only peripheral attachment to the labour market. The latter can be identified as parents who were not employed for most of the 48 months before the current spell of unemployment. Together with our focus on benefit receipt, our data might thus provide a conservative bound for the effects of the timing of parental unemployment. Effects might prove even starker among children whose parents have more precarious work histories and less access to the benefit system.

**Table 1.** Descriptive statistics (means/proportions) for each population in this study.

	Sorting in secondary school		Postsecondary enrolment	
	Paternal unemployment	Maternal unemployment	Paternal unemployment	Maternal unemployment
Girl	.49	.49	.52	.52
Born in the Netherlands	.94	.96	.95	.96
Twin birth	.03	.03	.03	.03
Birth order	1.4	1.4	1.4	1.4
Parental education: Degree	.37	.32	.57	.52
Household type: Cohabiting couple	.12	.12	.09	.09
Household type: Married couple	.83	.63	.86	.70
Household type: Single parent	.05	.25	.05	.21
Parental wealth: Below 2006 median	.63	.65	.50	.53
Parental employment, last 48 months: Continuous (v. intermittent)	.77	.81	.75	.72
Parent received (*q*−1)				
Social assistance	.004	.01	.003	.005
Sickness/disability benefit	.05	.06	.01	.02
Other social security	.01	.004	.004	.002
*N*	44,637	43,851	38,328	38,216

We will also consider heterogeneous effects based on parental wealth. We follow the bulk of research on wealth stratification (Killewald et al. 2017) and measure parental wealth as net worth, the sum of the value of household assets minus the value of debts. Whenever values differ between partners, we take the average between the two. To ensure enough cell size for our identification strategy, we split the sample based on the median net worth value; alternative splits will be presented in our sensitivity analyses. To net out changes in the distribution of wealth over time (e.g. due to the recession), we anchor the median to its value in 2006 deflated at 2015 prices (116,650 EUROS). This median value is taken from the wealth distribution of all parents of children in the birth cohorts of interest. Children are assigned to the low- and high-wealth group based on the value of parental wealth at the beginning of the year when parental unemployment occurred. This should minimise the chances that our measure of wealth might be affected by parental unemployment and thus lead to post-treatment bias. Sensitivity checks will assess whether our results change depending on such choices.

### 3.2. Identification and estimation: capturing the effects of the *timing* of parental unemployment

The timing of parental unemployment is the exposure in our study. We expect that spells of parental unemployment will be particularly detrimental for sorting across secondary-school tracks if occurring before the school advice and, in most schools, around the time of the CITO test. Parental unemployment in schools that do not administer the CITO might still influence children's performance and grades, as well as teachers' perception of the student. Given the quarterly frequency of unemployment data, we can define the timing of parental unemployment based on quarters relative to testing (for some) and the school advice for all children. We thus define *treated* children as those exposed to parental unemployment in the first quarter (January to March^3^) of the calendar year corresponding to 6th grade, henceforth the ‘winter’ quarter.

We compare these children to those exposed to parental unemployment in 6th grade but in the subsequent quarter of the same calendar/school year (April to June), henceforth the ‘spring’ quarter. By then, disruptive events such as parental unemployment are unlikely to affect the school advice. Further, by experiencing parental unemployment in a matter of days or a few months after the winter quarter, children in the spring quarter will likely be similar to their counterparts in many ways except for the timing of parental unemployment.

Focusing on students in 6th grade in 2008 for illustrative purposes, Table A1 in the Appendix supports this intuition. Children in the ‘winter’ and ‘spring’ group are largely comparable across various socioeconomic and family background factors that may affect both parental unemployment risks and children's education. Normalised differences across covariates are well within the bounds typically taken to signal good balance in the literature (±0.25, Imbens and Wooldridge 2009). In Figure A3 in the Appendix (top panel), we repeat the same exercise for the same covariates and all the main combinations of grade and year in our data, finding small normalised differences across the board.

Figure A3 thus provides reassurance on the absence of measured sources of confounding bias. As for unmeasured confounding, any unmeasured difference between the ‘winter’ and ‘spring’ group should be orthogonal to children's school grade. A sector-specific seasonal shock affecting parents only in one of the two quarters, for example, will likely affect parents of children attending 6th grade similarly to those attending other grades. For those experiencing parental unemployment in grades other than 6th grade, absent the causal mechanism working through the school advice, any outcome difference might thus be imputed to unmeasured confounding, e.g. quarter-specific shocks. Contrasts between the ‘winter’ and ‘spring’ group in grades other than 6th grade serve, in other words, as placebo checks or, more specifically, negative controls (Lipsitch et al. 2010), i.e. contrasts for which causal effects are expected to be null due to the absence of the causal pathway (through the school advice), but that may help detect and net out confounding bias instead.

We use the following linear model to estimate causal and non-causal comparisons of secondary-school track enrolment chances among children of the unemployed:

(1) $$yi=\alpha +\sum_{q}\sum_{g=-3}^{g=2}\beta g,q\cdot \mathrm{Unemployment}i,g,q+Xi\delta +\lambda g+\theta t+\epsilon i$$

where yi takes value 1 when a child *i* is enrolled in either HAVO or VWO by the third year of secondary school and is 0 otherwise (VMBO). To buttress our analyses of track placement, we also present results where yi is the CITO test score in 6th grade, a continuous outcome ranging from 501 to 550. For this outcome, we rely on Equation (1) to estimate effects at the mean and quantile treatment effects, using residualised quantile regression (RQR) for the latter (Borgen et al. 2022).

In the study population, children can be exposed to parental unemployment in either of two quarters *q* (winter and spring) and across different grades *g*, with *g* = 0 representing 6th grade. Hence, the difference between estimates of βg=0,q=winter and βg=0,q=spring provides us with the contrast of interest, the average treatment effect on the treated (ATT) by a spell of parental unemployment timed around the school advice. As for negative controls, we include three grades before 6th grade, i.e. the central grades of primary school, and two grades after, i.e. the first two years of secondary school (for details on a similar application, see Fradkin et al. 2019). In the following, we present our estimates normalised to the grade before treatment (5th grade, *g* = −1) to ease comparisons and with the aim of netting out unmeasured confounding.

The model includes a vector of covariates measured at the child, parent, and household level. These variables are, respectively, (a) dummies for sex of the child, whether the child was born in the Netherlands or abroad, birth order (top-coded for 3rd and higher-order parities), twin birth, and month of birth; (b) separate dummies for whether the parent received social assistance, sickness benefits or other social security benefits in the quarter before unemployment, a dummy for parental work experience (0 if a parent works continuously in the 48 months before unemployment, 1 if employed with gaps), dummies for parental birth cohort; (c) a dummy for household net worth (1 if below the median), one dummy for whether at least one parent holds a tertiary-education degree, and dummies for family structure in the quarter prior unemployment (married, cohabiting, single parent). In the likely absence of measured confounding (as per, e.g. Figure A3), these covariates are included to improve the precision of the estimation. The same applies to grade fixed effects λg (Fradkin et al. 2019). Year fixed effects θt help us focus on children exposed to parental unemployment in different quarter-grade combinations but within the same calendar/school year.

Throughout, point estimates are accompanied by robust standard errors.^4^ For inference, each individual result should be judged on its own to reject the overall null. Specifically, if the overall null is that there is no true treatment effect, rejection of the null requires that (a) the treatment effect must be significant, (b) each placebo estimate should be close to zero and non-significant and (c) statistically different from the treatment. This setting differs from one in which researcherscould reject the overall null if at least one (any) estimate among many reaches statistical significance and where, hence, a correction for multiple comparisons is warranted (e.g. Rubin 2021).

We follow the same strategy when evaluating the effect of the timing of parental unemployment on postsecondary school enrolment. We maintain the same assumption of unconfoundedness based on comparing children exposed in adjacent quarters of a treatment year and the same quarters in negative-control years. In line with the institutional features of the Dutch educational system, we expect both the spring and ‘summer’ quarters (hence, April to September) in a student's graduation year to be decisive for postsecondary enrolment decisions. We select a sub-population such that children in the spring-summer group are compared to those exposed to parental unemployment in the subsequent ‘fall’ quarter (October to December), to keep our focus as much as possible on adjacent quarters. Except for a handful of outliers, Figure A3 (bottom panel) in the Appendix shows small normalised differences between these two groups across all relevant covariates in all grade-by-year combinations. Once again, we include negative-control grades and normalise our estimates to the year before graduation.

To grasp the role of insurance mechanisms, we first conduct separate analyses for families below and at or above median parental net worth. We then add an interaction between unemployment timing and the monthly amount (EUROS, 2015 prices) of unemployment benefit entitlements received by the parent in question. As a result, we can evaluate whether families with lower wealth benefit more from generous unemployment benefits than families with higher wealth. We expect that children will be less negatively affected by a badly-timed spell of unemployment when their parents receive more generous amounts, especially when private insurance (wealth) is low. To facilitate interpretations of the statistical interaction, we evaluate this interaction for children exposed in the treatment year (6th grade or high-school graduation year) at five percentile values (10th, 25th, 50th, 75th, 90th) of the distribution of unemployment benefit amounts in each population. All estimates are once again normalised with respect to the year before the treatment year. The corresponding monetary values are also reported.

## 4. Findings

### 4.1. The timing of parental unemployment and sorting in secondary school

**Figure 1.**
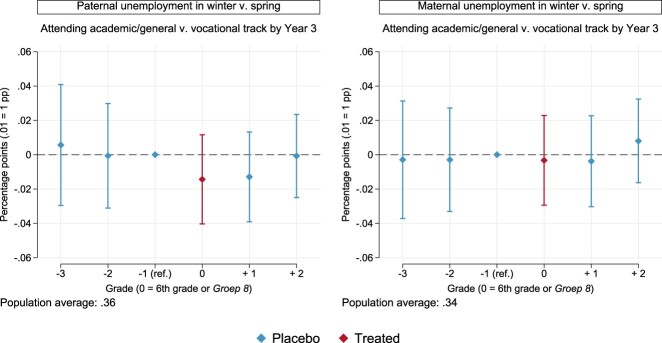
Point estimates and 95% confidence intervals for the effects of the timing of parental unemployment on the chances of attending academic/general v. vocational track by the third year of secondary school. Models are estimated as per Equation (1), with robust standard errors (see Table A3 in the Appendix).

Figure 1 provides estimates for all children in the populations affected by paternal and maternal unemployment around the time of secondary-school sorting. We display estimates in red for grades in which we expect ‘true’ treatment effects and in blue for ‘placebo’ grades (full estimates are presented in Table A3 in the Appendix).

We find no evidence of ‘bad timing’ on average. In the left panel, children exposed to paternal unemployment in the winter of 6th grade are around one percentage point less likely to attend the college/university-preparing tracks. Yet, the same can be said, for example, about children exposed in the winter of the first year of secondary school. Estimates are not statistically different from the reference in these two instances (*p* = .279 and *p* = .334, respectively) or in any other. There is little indication, overall, of a detrimental effect when paternal unemployment occurs in winter rather than spring and in 6th grade vis-à-vis any other grades. Similar considerations apply to children exposed to maternal unemployment, as per the right panel of Figure 1.

**Figure 2.**
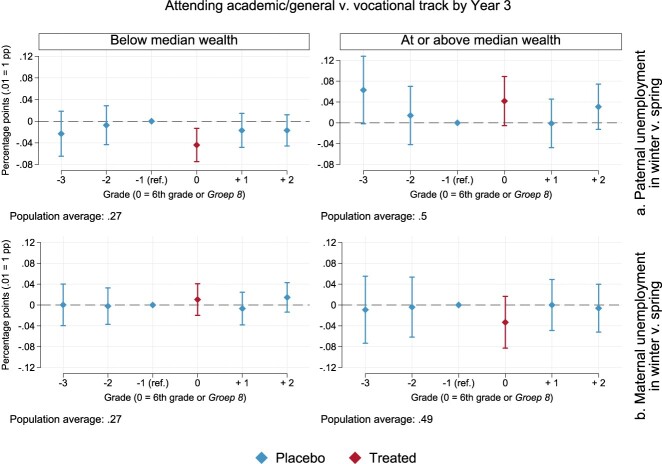
Point estimates and 95% confidence intervals for the effects of the timing of parental unemployment on the chances of attending academic/general v. vocational track by the third year of secondary school. Models are estimated as per Equation (1), separately by net worth at baseline and with robust standard errors (see Table A3 in the Appendix).

We then turn to possible heterogeneous effects. The top two panels of Figure 2 portray estimates for paternal unemployment (Table A3 in the Appendix). Among children with lower parental wealth, on the left, paternal unemployment seems most harmful if indeed timed around the school advice. Children exposed to paternal unemployment in the winter of 6th grade are around 4 percentage points less likely to attend a secondary-school track that prepares for higher education (*p* = .005). Roughly 27% of children from less well-off families attend academic/general tracks, and thus the treatment effect in 6th grade is estimated to reduce enrolment by roughly 15% (.04/.27×100).

If we were to normalise the treatment effect with respect to estimates in grades other than the 5th, contrasts would be similar in size, albeit occasionally more noisy.^5^ At the same time, we cannot detect any winter-spring difference in placebo grades, and point estimates in those years are closer to zero, as expected. Among children with higher parental wealth (top-right panel of Figure 2), the point estimate for 6th grade is positive and equals around 4 percentage points (p=.083). Yet, strong conclusions cannot be drawn due to the considerable overlap between interval estimates in the treatment grade and three out of four placebo estimates for this group of families. The same uncertainty holds for both well-off children and their less well-off counterparts when exposed to maternal unemployment, as displayed in the bottom two panels of Figure 2. On balance, these first estimates are suggestive of a negative effect of paternal unemployment around the school advice, albeit only among families with lower wealth.

**Figure 3.**
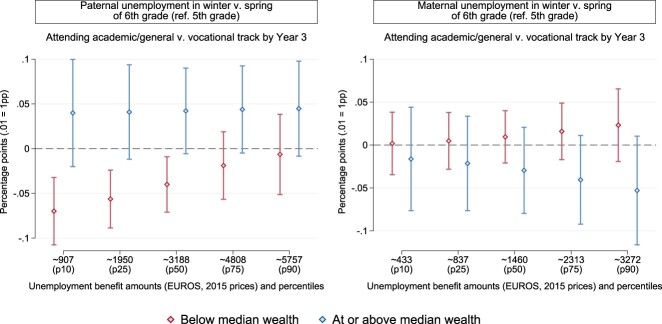
Point estimates and 95% confidence intervals for the differential effects of the timing of parental unemployment in 6th grade by unemployment benefit amounts. The outcome is the probability of attending academic/general v. vocational track by the third year of secondary school (ref. 5th grade). Models are estimated as per Equation (1), plus an interaction with unemployment benefit amounts, separately by net worth at baseline and with robust standard errors (Table A5 in the Appendix).

To grasp whether and to what extent the role of parental wealth is modified by public provisions, we estimated models as per Equation (1) separately by parental wealth and adding an interaction between unemployment timing dummies and the amount of unemployment benefits. In Figure 3 (Table A5 in the Appendix), we focus on results for the treatment year, i.e. when children are in 6th grade. A clear interaction between public and private insurance is found for paternal unemployment (left panel). Similar to average effects by parental wealth in Figure 2, we only detect negative effects of paternal unemployment around the school advice in families with lower wealth at baseline. Further, effects are larger the lower the amount of unemployment benefits allotted to children's fathers (*p* = .022 for the statistical interaction between paternal unemployment in 6th grade and benefit amounts, normalised against the corresponding 5th-grade estimate). Children's chances of being enrolled in the academic/general tracks of Dutch secondary school are estimated to decrease by 7 percentage points (*p*<.001) if fathers have the lowest entitlements (≈907 EUROS per month) in families below median wealth. The negative effect reduces, assuming linearity, to 6 percentage points at the second-lowest benefit amount (*p* = .001) and to 4 points at the median value of unemployment benefits for fathers in our population (*p* = .011, ≈3,188 EUROS per month). At larger levels of the entitlement, estimates shrink further (−2 pp, *p* = .327 at the 75th percentile and −1 pp, *p* = .777 at the 90th). No such pattern is detected among children in families at or above median wealth (and *p* = .876 for the statistical interaction between paternal unemployment in 6th grade and benefit amounts, normalised against the 5th-grade estimate). Although more noisy, and often of comparable size with estimates for fathers, we cannot detect meaningful differences when examining the timing of maternal unemployment and treatment effect heterogeneity by wealth and benefit amounts (Figure 3, right panel).

Hence, among families with lower wealth, estimates suggest a moderate negative effect of paternal unemployment timed around the school advice in 6th grade on children's chances of attending secondary-school tracks that prepare them for higher education in the Netherlands. In the absence of effective private insurance, such consequences of paternal unemployment in families with lower wealth are mitigated more strongly by public insurance in the form of unemployment benefits. Assuming linearity, the negative effect of a badly-timed spell of paternal unemployment is more than halved when comparing the 10th and the 75th percentile of benefit entitlements in lower-wealth families. The negative effects of paternal unemployment in 6th grade are moderate in size for a large fraction of affected children in families with lower wealth, including those with median benefit amounts.

### 4.2. The timing of parental unemployment and enrolment in postsecondary education

**Figure 4.**
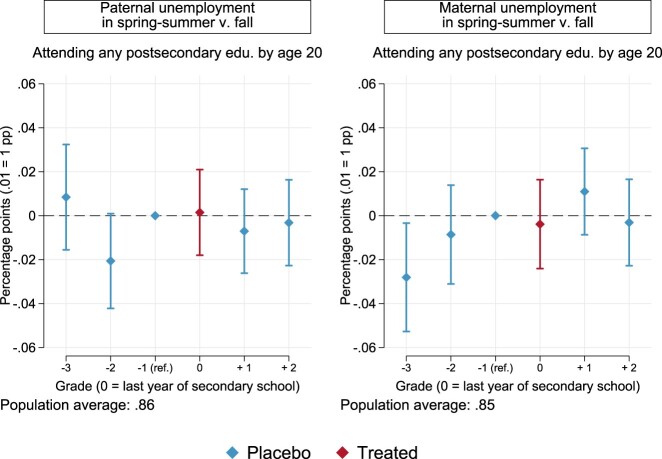
Point estimates and 95% confidence intervals for the effects of the timing of parental unemployment on the chances of attending any postsecondary education by age 20. Models are estimated as per Equation (1), with robust standard errors (see Table A4 in the Appendix).

As for post-secondary enrolment, we examine average differences depending on the timing of paternal and maternal unemployment. In the left panel of Figure 4 (for full estimates, Table A4 in the Appendix), we cannot detect any treatment effect of paternal unemployment occurring in the spring and summer of graduation year, as opposed to the fall of the same year (*p* = .882) and when further compared to the same seasonal contrast one year before graduation. Similar conclusions can be drawn for maternal unemployment around graduation (*p* = .709). Likewise, comparing estimates for graduation year with other placebo grades in both populations, as well as estimates across each grade within each population, we find little evidence of differential effects when confidence bands are also taken into account. All in all, timing within the school year seems not to matter differentially across grades when it comes to parental unemployment and post-secondary education, at least on average in the populations under study.

**Figure 5.**
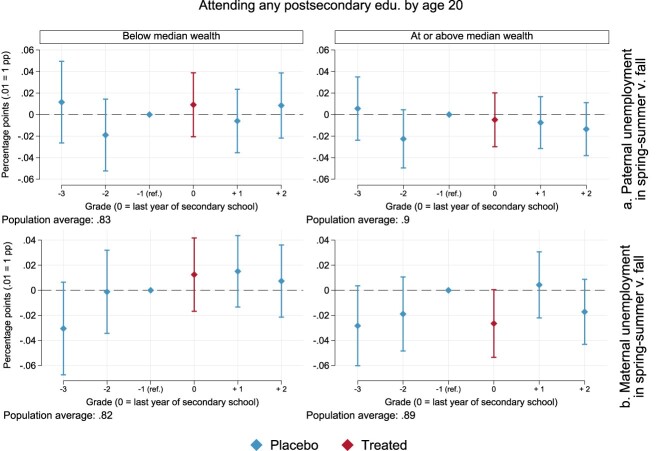
Point estimates and 95% confidence intervals for the effects of the timing of parental unemployment on the chances of attending any postsecondary education by age 20. Models are estimated as per Equation (1), separately by net worth at baseline, and with robust standard errors (see Table A4 in the Appendix).

When we turn to heterogeneity by parental wealth, differently from the case of sorting in secondary school, we cannot detect any clear-cut pattern. For paternal unemployment at the top of Figure 5 (Table A4 in the Appendix), we find that treatment effects in graduation years are very close to zero, both among families below median wealth and for those at or above the median. When also compared to grades other than the chosen reference group in the year before graduation, we cannot appreciate statistically or substantially meaningful deviations in the treatment year. The same holds for maternal unemployment at the bottom of Figure 5, particularly in families with lower relative wealth. Among more well-off families, children exposed to maternal unemployment around high-school graduation appear to be 3 percentage points less likely to attend any post-secondary education by age 20 (*p* = .054), once again when normalised to the preceding placebo year. Yet this estimate is hardly distinguishable from the ones in most of the other placebo years. For one, contrasting graduation year and the placebo estimate for two years prior yields a *p*-value of .615, and point estimates are roughly the same. We do not find robust evidence, in sum, that timing of parental unemployment within the school year matters across the selected grades, when it comes to future chances of post-secondary enrolment in families across the wealth divide.

**Figure 6.**
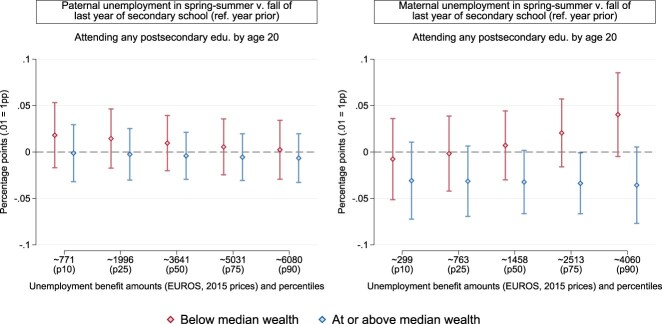
Point estimates and 95% confidence intervals for the differential effects of the timing of parental unemployment in 6th grade by unemployment benefit amounts. The outcome is the probability of attending any postsecondary education by age 20. Models are estimated as per Equation 1, plus an interaction with unemployment benefit amounts, separately by net worth at baseline and with robust standard errors (see Table A6).

If not due to private insurance *per se*, the timing of parental unemployment might have different consequences depending on the interaction between private and public insurance. Figure 6 (Table A6 in the Appendix) displays findings for separate models by parental wealth, adding an interaction between the timing of parental unemployment and unemployment benefit amounts. We focus on the treatment year, the last year of secondary school. Different from previous results for secondary-school enrolment, we do not find evidence of an interplay between public and private insurance. Estimates for both paternal unemployment are positive at most levels of unemployment benefits in families with lower wealth, close to null in families with higher wealth, and in all cases statistically indistinguishable from zero and from each other. Exposure to maternal unemployment in wealthier families is associated with a fairly stable penalty of around 3 percentage points across all levels of unemployment benefits. As in our main analyses, however, these estimates are sensitive to the grade we select for normalisation and, therefore, we refrain from strong conclusions.

There is little indication, hence, that the timing of parental unemployment matters *within* school years when it comes to chances of attending postsecondary education in the Netherlands.

## 5. Additional analyses

### 5.1. Mechanisms

Evidence in Figure 2 suggests that unemployment might adversely affect less well-off children in 6th grade. Lower test performance, either due to stress or lower parental investment in shadow education, could contribute to explaining this finding. We thus repeat our analyses split by parental wealth and examine CITO test scores. We observe CITO scores for around two-thirds of the students in our study (Np=28,628; Nm=28,100), as around one third of schools do not engage in testing within the CITO framework.

Results in Figure A4 in the Appendix provide some evidence concerning adverse effects on school performance at the mean. For families below median wealth in the top-left panel, we find that paternal unemployment in winter rather than spring of 6th grade reduces CITO scores by around 1 point (10% of a SD, *p* = .019), compared to the reference. For more well-off families, on the other hand, the estimate stops at −0.12 (*p* = .811). No other contrast, including for maternal unemployment at the bottom of Figure A4, is statistically different from zero. However, considerable overlap exists between interval estimates across grades and sub-populations. Therefore, we only regard these results as suggestive of a negative effect of paternal unemployment timed around the 6th-grade test on children's test performance in families with lower relative wealth.

We then consider the interaction between private and public insurance and test scores, focusing on children exposed to paternal unemployment in the winter rather than the spring of 6th grade. Similar to Figure 3, Figure A5 in the Appendix shows clear evidence of detrimental effects on children's test performance for those *both* below median wealth and with the lowest benefit entitlements. Linear predictions point, at worst, to a reduction in test performance by 1.8 points (*p* = .001) for children whose fathers are in lower-wealth households and with the lowest benefit entitlements and 1.4 points in the following group (*p* = .003). Both estimates are somewhat larger than the average effect in Figure A4. The figure closely mirror Figure 3 for track choice, with estimates close to zero for the higher-wealth group. Altogether, estimates suggest that lower test performance may be part of the mechanism driving down children's education chances after a badly-timed spell of paternal unemployment in families with lower wealth.

Beyond effects at the mean, we also investigate whether the timing of parental unemployment is detrimental at specific cutoff points of the test-score distribution. The top panel of Figure A6 in the Appendix presents descriptive findings and quantile treatment effects (QTE) for children in lower-wealth families. The effects of paternal unemployment in the winter of 6th grade are somewhat concentrated at the beginning of the distribution, particularly around the 40th percentile of the distribution (QTEp40=−1.4 points, *p* = .006; CITOp40≈530). We can speculate that, counterfactually, students might have scored high enough to receive a ‘mixed’ VMBO-HAVO advice and eventually enrol in HAVO, if not for the timing of paternal unemployment. Nevertheless, estimates are often statistically indistinguishable from one another along the distribution (e.g. *p* = .807 for QTEp40 = QTEp90). To provide further guidance, we also investigate the chances of being enrolled in HAVO separately from those of enrolling in VWO (Figure A8 in the Appendix). In line with our results concerning CITO scores, we find somewhat larger and more clear-cut penalties with respect to enrolment in HAVO (−3 pp, p=.030; for VWO: −2 pp, *p* = .179).

For children in the higher-wealth population, we can detect effects higher up in the distribution, e.g. around the 80th percentile (QTEp80=−.9 points, *p* = .011). This corresponds to a score of around 545, the cutoff point for VWO. Once again, the pattern along the distribution is not clear-cut. We repeated our main analyses separating enrolment in HAVO and VWO for children at or above median wealth too. We find that, despite some evidence of lower scores around the cutoff point for VWO, there is little evidence of a decrease in the chances of attending VWO for children whose father experienced unemployment in the winter of 6th grade in higher-wealth families.

For completeness, we also present quantile analyses for maternal unemployment (Figure A7 in the Appendix). Results are largely inconclusive, mirroring the findings at the mean. Separate analyses for the chances of attending HAVO and VWO also do not reveal a clear-cut pattern (Figure A9 in the Appendix).

### 5.2. Robustness and sensitivity checks

*Long-run effects of paternal unemployment in 6th grade*. – By affecting secondary-school track enrolment in a stratified system like the Dutch one (Figure A1), the timing of paternal unemployment in 6th grade may have long-run consequences on children's postsecondary education too. We explore these long-run effects in Figure A10 in the Appendix. In families with lower than median wealth, and keeping the 5th-grade estimate as our baseline, we find that paternal unemployment in the winter of 6th grade reduces the chances of enrolment in postsecondary education by around 2 percentage points by age 20, although *p* = .293. Other contrasts are more conclusive (e.g. – 4 percentage points taking the first year of secondary school as a baseline, *p* = .012). Overall, however, long-run estimates in Figure A10 do not decisively mirror short-run estimates on track placement.

*Parental wealth groups*. – We also revisit heterogeneous effects by asking if the split at the median reflects a wealth gradient, i.e. negative effects monotonically increasing as parental wealth declines, or if certain groups such as those with the lowest wealth among the least well-off drive our estimates. Hence, we investigate narrower wealth groups divided by quartiles of the distribution of parental net worth. Figure A11 in the Appendix shows that, for paternal unemployment in 6th grade, effects are driven by families below median wealth *and* above the first quartile. In this group, children affected by paternal unemployment in the winter (v. spring) of 6th grade are around 7 percentage points less likely to attend the general/academic track by the third year of secondary school (*p* = .006). Their performance at the CITO also decreases the most (−1.85 points, *p* = .004). They are also 6 percentage points less likely to be enrolled in postsecondary education by age 20 (*p* = .023). Hence, our main findings concerning paternal unemployment in 6th grade appear driven by children in the second quartile group, for whom short-run effects on track placement and test scores translate into long-run penalties in terms of postsecondary enrolment. Differently, other results in Figure A11 do not add new information compared to our main analyses split around median wealth.^6^

*Parental wealth definition*. – Our wealth measure is based on net worth, taking the 2006 median value from *all* parents of children in relevant birth cohorts. We assess here if our results are unchanged when taking the median from the final sub-population of parents or without anchoring to the 2006 value. We also consider more composite measures of parental wealth, limiting our analyses to children below the (2006, anchored) median of both net worth and savings, to gauge the importance of liquidity, and looking at families with low net worth and either no mortgage debt or high mortgage debt, to examine the role of housing wealth. Finally, we contrast our analyses for families with lower wealth to those performed among families with lower parental education, i.e. in which no parent holds a postsecondary degree. For reference, we also include estimates for families where at least one parent holds a degree. Parental education has often been examined in the field of parental unemployment, at times alluding to its links with parental wealth (Lindemann and Gangl 2020), and it is most often conceived as the main stratifying force behind children's education, especially in the Netherlands (e.g. De Graaf et al. 2000; Inspectorate of Education 2016).

We compare average effects of paternal unemployment in the winter of 6th grade on sorting in secondary school in Figure A12 in the Appendix. Our choices regarding net worth are largely inconsequential. We obtain comparable estimates regardless of which median value is considered. When incorporating savings in the definition, estimates are also statistically indistinguishable but reduce somewhat to a 3 pp penalty (*p* = .044). We also obtain comparable point and interval estimates when considering low-wealth households with either no mortgage debt (−4 pp, *p* = .031) or high mortgage debt (−4 pp, *p* = .050). If we had stratified our analyses by (a coarse measure of) parental education, instead, we would not have detected a negative effect of paternal unemployment timed around the school advice. Estimates in families with lower and higher formal education are much closer to 0 than those obtained when stratifying by parental wealth, and are both statistically indistinguishable from zero and from each other (−2 pp, *p* = .299 and −1 pp, *p* = .759, respectively).

When we turn to the interaction with public insurance, the gradient observed in Figure 3 holds, by and large, regardless of which wealth-based definition of private insurance we use. As per Figure A13 in the Appendix, the gradient is only somewhat less steep when singling out families who are below the median for both net worth and savings, and similar estimates are obtained for families with lower net worth regardless of mortgage debt. Once again, when switching from wealth to parental education, we cannot detect clear-cut effects nor differences between households depending on parental education at different benefit levels. Wealth, hence, appears to stratify the effects of the timing of parental unemployment to a larger and clearer extent than parental education in our setting. All together, our main findings hold regardless of the operationalisation we use for net worth. Negative timing effects are not driven by families with lower liquid wealth (savings).

*Endogeneity of unemployment benefits*. – Receiving unemployment benefits is endogenous to the experience of unemployment, a ‘post-treatment’ variable that, once conditioned on, might bias estimates (Elwert and Winship 2014). Given that our treatment is the *timing* of parental unemployment, however, we should be concerned about post-treatment bias only if benefit amounts differed between parents who entered unemployment, say, in the winter of 6th grade versus the spring of the same grade, and more so in that grade as opposed to any other grade (e.g. the reference in 5th grade). Such differences would suggest the presence of unmeasured confounding, i.e. that families exposed to unemployment in different quarters and grades also differ for some unobserved reasons (luck, seasonal shocks, policy changes, ability, genes, etc.), which then lead parents to receive different benefit amounts – and perhaps also drive differential educational outcomes for their children. In Appendix 6, we show that this is unlikely, as we cannot find any evidence of differences in benefit amounts depending on the timing of parental unemployment (Figure A14, left panel). As a further check, we also show that parents in our setting do not differ in the duration of their unemployment spells, across quarters and grades (Figure A14, right panel). In other words, our empirical strategy assures that families differ in the timing of unemployment, as well as the timing of unemployment benefits, but not with respect to the (confounding) drivers of unemployment itself or of benefit amounts and unemployment duration.

## 6. Discussion

We have studied whether, to what extent, and for whom the timing of parental unemployment can shape educational trajectories. We find five main results. First, children exposed to parental unemployment around the time of a high-stakes test in 6th grade are less likely to be enrolled in the college/university-preparing tracks of secondary school, but only when paternal unemployment^7^ comes in families with lower wealth. Second, limited to less well-off families, the effects of bad timing increase when fathers receive lower unemployment benefit amounts and are mitigated vice versa by larger amounts – particularly if above the median benefit. Third, such effects partly manifest via school performance in a high-stakes test administered in most schools in 6th grade. Fourth, we show that children in families with lower wealth, especially those in the second quartile group, are also less likely to attend any postsecondary education by age 20 when affected by paternal unemployment in the winter of 6th grade. Last, our results provide little indication that spells of unemployment around the end of secondary school alter children's chances of attending postsecondary education by age 20.

With our first contribution, we show that the timing of parental unemployment can affect educational transitions much earlier than by the time of children's postsecondary enrolment. Results in this study may spur research in contexts with similar school tracking or ability grouping in Europe (see also, Schmidpeter 2020) and inform debates about the inequalities therein (e.g. Hanushek and Wößmann 2006; Van de Werfhorst 2019). Evidence on lowered test performance also bears relevance for debates on (high-stakes) testing (e.g. Buchmann et al. 2010; Zwier et al. 2020; Högberg and Horn 2022). We add to these debates by providing an example of the mechanism through which socioeconomic disadvantage (here, low wealth) can affect children's test performance, i.e. by limiting families' insurance against hardships such as parental unemployment (Pfeffer 2011; Kalil and Wightman 2011). As per our Background, such vulnerability might be due to heightened stress or lower investments in shadow education. The first limitation of our analyses is that we cannot observe these mechanisms directly. Both stress and investments might be particularly affected when fathers enter unemployment in the Netherlands, given men's typical role as primary earners within the Dutch ‘one-and-a-half’ breadwinner model. Future research could investigate how insurance unfolds via stress, investments, or other channels across families and contexts.

In line with a growing body of literature (Coelli 2011; Pan and Ost 2014; Lehti et al. 2019; Schmidpeter 2020), our second goal has been to complement earlier research on the effects of parental unemployment *per se*, turning attention to its timing relative to children's life course. In this respect, effect sizes are comparable to previous studies. Around the tracking decision, we find an average penalty of 4 percentage points on the chances of enrolling in general or academic tracks associated with paternal unemployment among less well-off families in the Netherlands. Oosterbeek and colleagues (2021) study a different ‘timing-based’ treatment in the Dutch context, that of children's school-starting age, and find similarly sized effects on track placement. When examining unemployment around tracking in a similar institutional setting (Austria), Schmidpeter (2020) finds a 4–5 percentage-point penalty (16% of baseline), this time on children's chances of university degree attainment.

As for unemployment around high-school graduation, finding small and null effects accords with only part of the literature. As documented by Hilger for the US (Hilger 2016), it could be that the financial losses attached to unemployment are such that prospective students fall below the thresholds for more generous student aid, thus maintaining their enrolment chances intact.^8^ Alternatively, the fact that parental unemployment's timing might not adversely affect postsecondary enrolment might be a by-product of the previous sorting in college- and university-preparing tracks. Students ‘at risk’ of accessing higher education in the Dutch system could be a selected group (also) when it comes to their response to shocks like parental unemployment.

Another limitation of our findings is that they cannot be directly interpreted in terms of gaps between children with unemployed and employed parents. Compared to well-established research on the latter gap, we focus on a different estimand (Lundberg et al. 2021) by asking if, among children of unemployed parents, some are even more penalised than others^9^ based on the timing of unemployment. Prior research has often defined timing in terms of children's age, whilst we highlight the merits of disaggregating timing relative to the institutional features of the educational system. Future research could rely on even more precise timing definitions relative to testing or educational transitions, whilst we were bound to the quarterly frequency of our unemployment data. Similar designs could also be used to grasp the intergenerational effects of adversities such as parental divorce or health shocks due to illness or death within families (see, e.g. Stans 2022). The timing of such shocks might have gained new relevance during the COVID-19 pandemic, compounding or perhaps partly driving the effects on children's education attributed to remote learning during school closures (Engzell et al. 2021). Future research, also within the context of the pandemic, could thus build on the timing-of-events and negative-control approach followed in this paper.

Finally, findings provide a novel example of the insurance function of wealth for children's education (Pfeffer 2011; Killewald et al. 2017; Pfeffer 2018), and of its interplay with public insurance offered by unemployment benefits. Our findings expand on previous studies for the US showing that wealth might, when lacking, exacerbate the intergenerational effects of unemployment^10^ (Kalil and Wightman 2011; Pan and Ost 2014). Such prior literature relied on proxies of wealth such as homeownership, whilst we have measures of net worth, most commonly used in studies on wealth disparities (Killewald et al. 2017), savings, and debt. Perhaps surprisingly, the sensitivity tests we conducted suggest that it is not this liquid component that drives wealth gradients in our study, nor that there are differences based on levels of household mortgage debt. It could be that families with lower net worth but above-median savings can sustain investments in shadow education, but are more prone to stress. For example, low net worth and no mortgage debt might equate to renting (Pan and Ost 2014), and thus the stress of living in more confined spaces, making rent, or frequent moves. In addition, the group driving our estimates might also consist of households with a mortgage that, especially due to the Great Recession in the period, exceeded the value of their property. The combination of the resulting indebtedness and relatively large assets is not uncommon in the Netherlands (Balestra and Tonkin 2018), and may leave households exposed to the stress related to changing circumstances such as unemployment (see also, Müller et al. 2021). Another possibility that we leave to further research is that differences by parental wealth in our study overlap to a certain extent with differences by ethnic/migrant background, given that large wealth gaps exist between White and minoritised communities in the Netherlands (e.g. Uunk 2017). Children from minoritised communities might be over-represented in the low-wealth group affected by parental unemployment in our study. As a result, whether the lack of private insurance is the only mechanism at play for all low-wealth families is an open question. For example, future studies using either qualitative or experimental methods could assess if teachers engage in ethnic-based discrimination and the latter explains part of the disadvantage faced by children in less well-off families hit by paternal unemployment (see, e.g. Inspectorate of Education 2016).

As for public insurance, evidence supports previous studies in equating insurance with the generosity of unemployment benefits (e.g. Gangl 2006; Sjöberg 2010; Lindemann and Gangl 2020). For paternal unemployment and sorting in secondary school (Figure 3) we have found a clear-cut gradient in the intensity of treatment effects depending on the size of allotted benefits. However, more generous benefit amounts also correspond to higher pre-unemployment earnings and human capital, which could also solve an insurance function by sustaining investments and outweighing stress and pessimistic outlooks. We should note that, when considering parental education as a proxy for human capital, we find no evidence of its insurance function in our study (e.g. Figure A12). Future research, nonetheless, could examine public insurance in settings where benefit entitlements do not depend on previous earnings or rely on reforms affecting the generosity of benefits whilst keeping earnings constant. Alternatively, to sort out whether earnings rather than benefits solve an insurance function, future studies could investigate whether parental re-entry in the labour market or an exogenous change to their earnings via wage rates (e.g. minimum wage legislation) might affect educational trajectories among children of ‘previously unemployed’ parents.

## Conclusions

We have shown that children's educational chances are responsive to the timing of paternal unemployment in less well-off families. Parental unemployment is consequential for educational careers and test performance when occurring around the time of sorting across secondary-school tracks in the Netherlands. In this respect, children in families with lower wealth have more to gain from more generous unemployment benefit entitlements. Despite recent retrenchment and extensive conditionalities (Mooi-Reci and Mills 2012), unemployment benefits in the Dutch system offer fairly extensive protections in comparative perspective (e.g. De Nardi et al. 2021). We can speculate that the effects detected in this study are a lower bound of the effects of ‘badly-timed’ spells of parental unemployment, which could prove even more sizeable in contexts with less generous out-of-work benefits or whenever households are ineligible for unemployment benefits. Our findings suggest that the intergenerational effects of parental unemployment could be tackled by policies targeting less well-off families right on time.

## Supplementary Material

Supplemental Material

## Data Availability

This paper uses administrative data from Statistics Netherlands (Central Bureau voor de Statistiek, CBS). The System of Social-statistical Datasets (SSD) microdata can be accessed via the following link: https://www.cbs.nl/en-gb/onze-diensten/customised-services-microdata/microdata-conducting-your-own-research. The SSD microdata was analysed via a secure internet connection (Remote Access) (https://www.cbs.nl/en-gb/our-services/customised-services-microdata/microdata-conducting-your-own-research/rules-and-sanctioning-policy) after receiving authorization from Statistics Netherlands (CBS). For further details regarding CBS microdata access, please send an email to: microdata@cbs.nl. Any errors or omissions are the sole responsibility of the authors. Replication codes can be found at https://osf.io/bc735/.

## Footnotes

1 The two years of pre-school preceding six grades of primary education are called *Groep 1* and *Groep 2* in the Dutch system, hence *Groep 8* coincides with 6th grade.

2 Some have reported a high correlation (.85) between test results and teacher advice (Borghans et al. 2020). The Inspectorate of Education, at the same time, has found that test results and teacher recommendations might systematically not coincide depending on the socioeconomic background of pupils. More often than their counterparts, those with high-income or non-migration backgrounds receive higher recommendations than those based on test scores alone (Inspectorate of Education 2016).

3 Except for sudden firm-level shocks (plant closures, downsizing, etc.), parents may have prior knowledge of their upcoming unemployment spell. As a result, stress might also build up in the preceding month(s). Compared to the control group, the treated in January-February will have such ‘anticipatory stress’ compounded by the experience of unemployment itself and the related financial and psychological consequences. As the CITO test is typically taken in February, test performance for children in the March group might be affected only by anticipatory stress and, as a result, be no different from that of children in the April group, for example. If anything, therefore, estimates of the treatment effect might be conservative due to the inclusion of the March group.

4 Working with population data, standard errors (SEs) do not reflect sampling-based uncertainty by definition. Rather, we present SEs to reflect ‘design-based’ uncertainty (Abadie et al. 2020), the fact that we cannot observe each unit's outcome under one intervention (e.g. being exposed to parental unemployment in winter) and under an alternative intervention (e.g. being exposed to parental unemployment in spring) at the same time. Abadie et al. (2020) have shown that robust SEs can be overly conservative in such causal designs when relying on population data, but we find that, in our setting, robust SEs are extremely close to those obtained via the correction they suggest (e.g. stratifying SEs by a third variable that could be the source of heterogeneous causal effects, such as calendar year or school grade in our study).

5 Subtracting estimates for grades other than the 5th to our 6th-grade estimate for paternal unemployment in families with lower wealth, we obtain the following *p*-values: pGrade6v.3=.312, pGrade6v.4=.035, pGrade6v.Year1=.071, pGrade6v.Year2=.047.

6 As for maternal unemployment, estimates in Figure A11 seem to indicate once again a detrimental treatment effect on educational outcomes in higher-wealth families. However, the estimates for mothers in the third or fourth quartile group are not distinguishable from most other placebo estimates (except, at times, the one used as a baseline). As such, results for maternal unemployment remain inconclusive.

7 Although beyond the scope of our study, we have also investigated differences in treatment effects for maternal unemployment when either excluding or including only single mothers. In both instances, we find null results indistinguishable from our main analyses (cf. Brand and Simon Thomas 2014).

8 Previous evidence shows that students are well-informed about student aid (Booij et al. 2012), which at the time comprised an income-based subsidy on top of a universal one. Whether and how circumstances might have changed in 2015, with the substitution of the universal grant with an income-contingent loan (van den Berg 2020), is an important avenue for further research.

9 In fact, as per Table A2 in the Appendix, children in the same birth cohorts whose parents never experience unemployment (claim unemployment benefits) have a more privileged background and better educational outcomes. This holds even for those in the lower-wealth group compared to their counterparts exposed to unemployment (cf., e.g. Figure 2). Average educational outcomes in the ‘never-unemployed’ population are more in line with those of children of unemployed parents in the higher-wealth group.

10 As our study focused on unemployment benefit claims, we could not tap into self-employed parents who, whilst possibly wealthier at baseline, may have lost their business due to bankruptcy, with possible spillovers on children's educational trajectory.
