# Supplementary material for: The timing of parental unemployment, insurance and children's education
Source: Eur Soc. 2023 Mar 22;26(3):602–38. doi: 10.1080/14616696.2023.2188550 (PMC11567677; doi:10.1080/14616696.2023.2188550)
Supplement: Supplemental Material [file REUS_A_2188550_SM5271.pdf]

The Timing of Parental Unemployment, Insurance and Children's Education
Appendix

Gabriele Mari[1] Renske Keizer[2] Ruben van Gaalen[3]

1. Dutch educational system

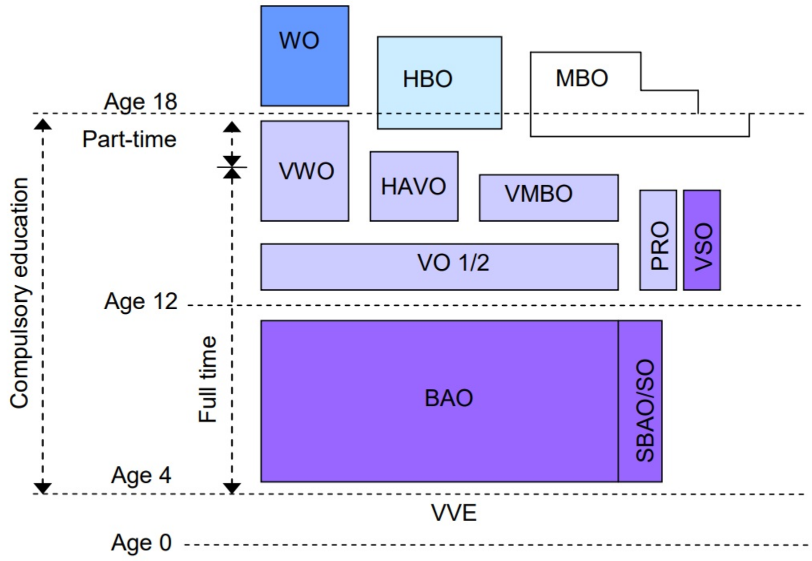

Figure 1A: Dutch educational system. VVE = Early childhood education; BAO = Primary education; SO = Special education; VO = Secondary education; PRO = Practical training; VMBO = Vocational secondary education; HAVO = General secondary education; VWO = Academic secondary education; MBO = Vocational upper-secondary education; HBO = Higher professional education; WO = University education. Note: Ages are only indicative (for more details, see Oosterbeek et al., 2021). Source: https://www.oecd.org/education/school/NLD_CBR_Evaluation_and_Assessment.pdf (last accessed: 16 December 2022).

[1]Erasmus University Rotterdam. Corresponding author: mari@essb.eur.nl.
[2]Erasmus University Rotterdam.
[3]Statistics Netherlands, University of Amsterdam (UvA).

2. Cell size

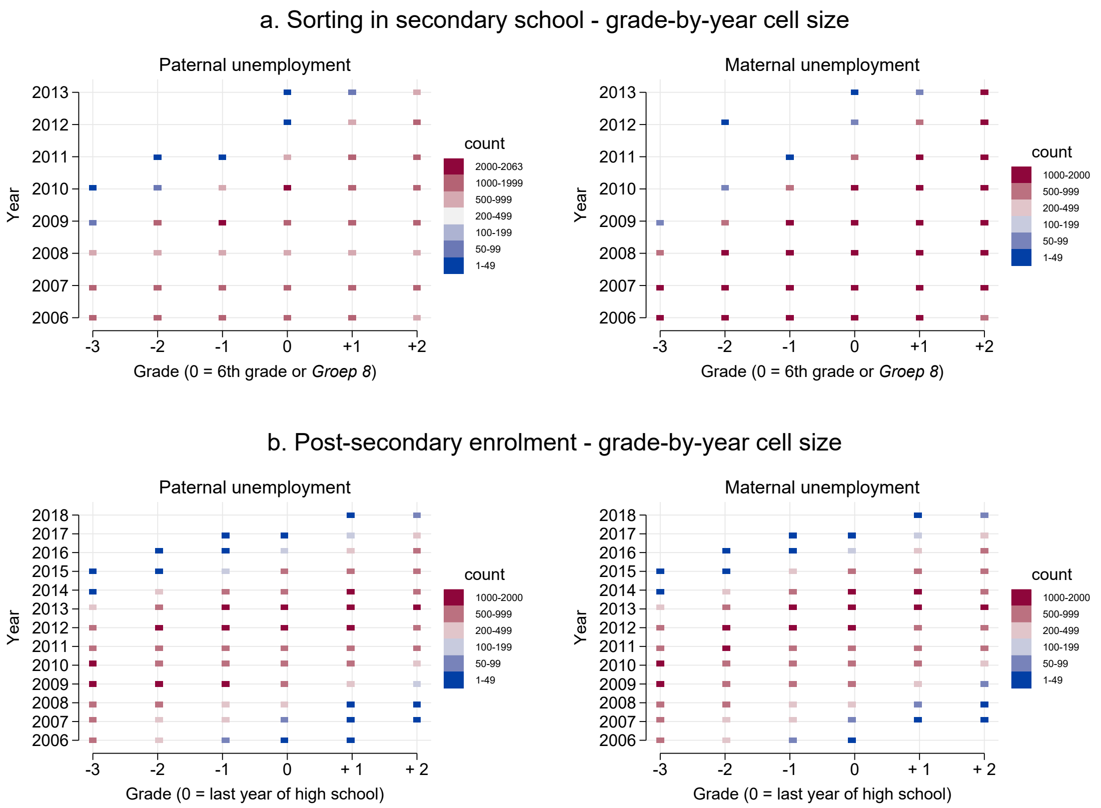

Figure 2A: Heat map of cell size in each grade by year combination. Own elaboration of CBS microdata, birth cohorts 1992-1998 exposed to parental unemployment in selected quarters.

3. Covariate balance depending on the timing of parental unemployment

Table 1A: Means/proportions for selected covariates (candidate confounders), children exposed to paternal unemployment in 6th grade, 2008.

	Paternal Unemployment	
	Winter	Spring
Parental education: Degree	.40	.41
Household type: Cohabiting couple	.10	.09
Household type: Married couple	.83	.86
Household type: Single parent	.07	.05
Parental wealth: Below 2006 median	.59	.60
Paternal employment, last 48 months: continuous	.74	.80
Father received (q - 1):		
Social assistance	.01	.01
Sickness/disability benefit	.04	.05
Other social security	.002	.002
n	493	407

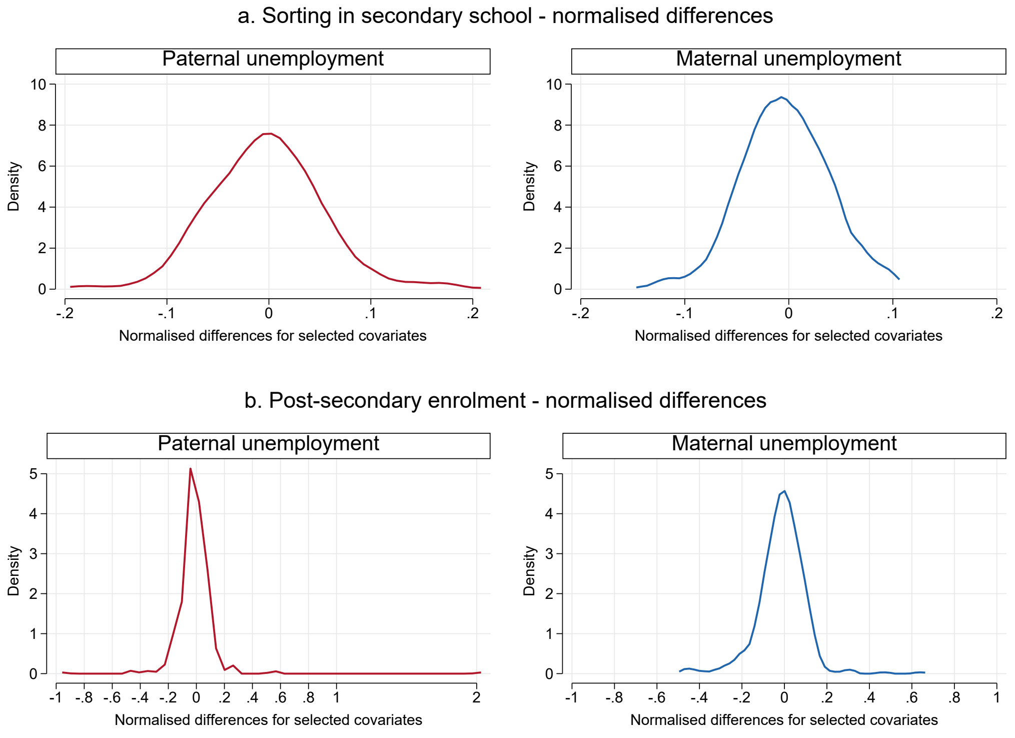

Figure 3A: Covariate balance depending on the timing of parental unemployment, across all candidate confounders as per Table 1 and for all grade-year combinations (with $n \geq 100$). Balance is expressed in terms of normalised differences (Imbens & Wooldridge, 2009).

4. The timing of parental unemployment and CITO test scores

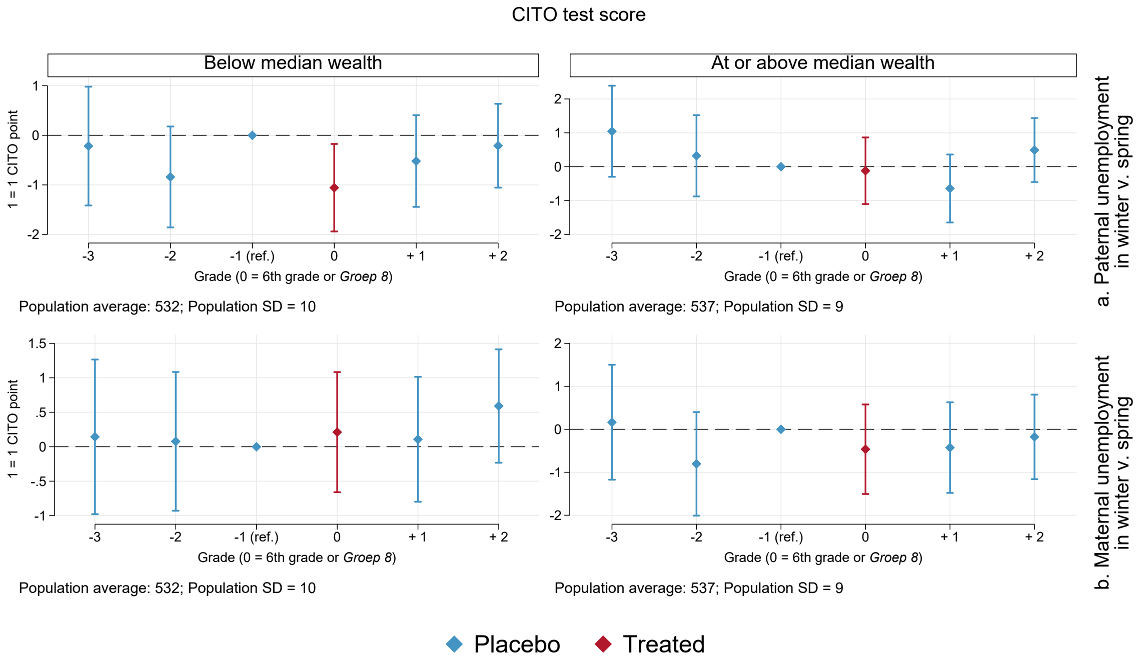

Figure 4A: Point estimates and 95% confidence intervals for the effects of the timing of parental unemployment on children's CITO test score. Models are estimated as per Equation 1, plus an additional control for whether Dutch is the main language spoken at home. Models are estimated separately by parental net worth at baseline and with robust standard errors.

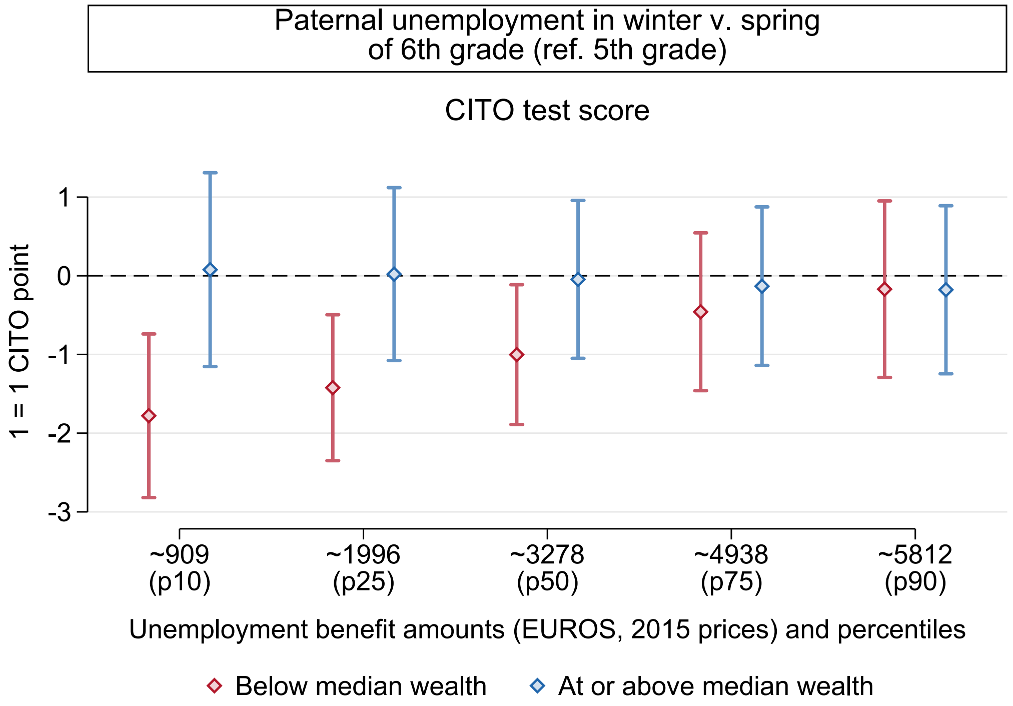

Figure 5A: Point estimates and 95% confidence intervals for the differential effects of the timing of parental unemployment in 6th grade by unemployment benefit amounts (normalised to the corresponding 5th-grade estimates). The outcome is children's CITO test score. Models are estimated as per Equation 1, plus an additional control for whether Dutch is the main language reported at home. Models include an interaction with unemployment benefit amounts, and are estimated separately by parental net worth at baseline and with robust standard errors.

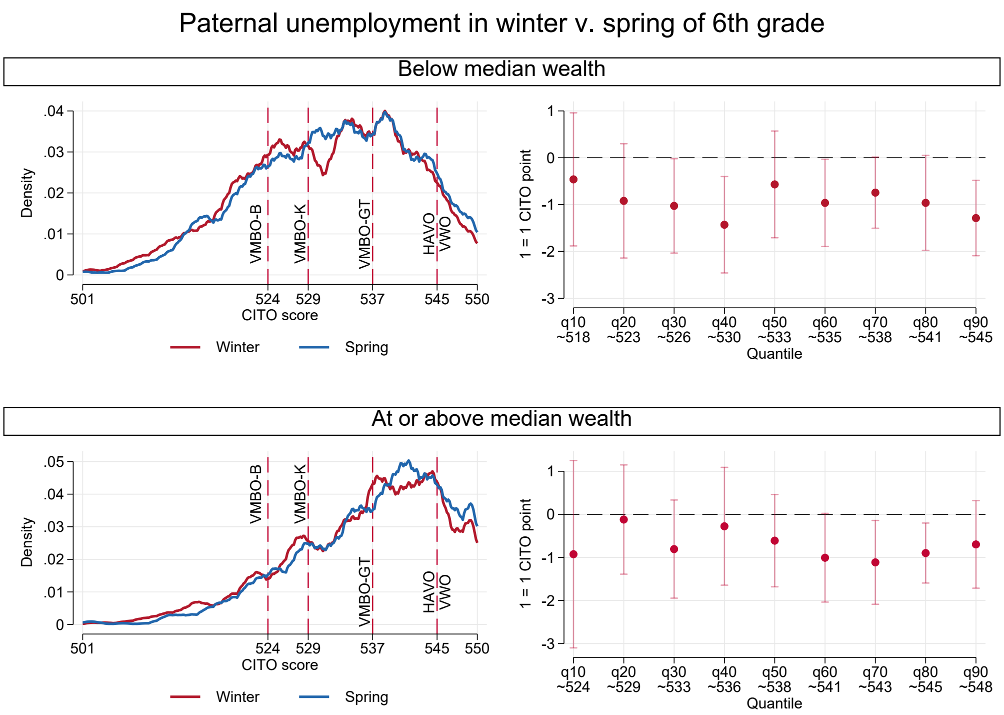

Figure 6A: Timing of paternal unemployment and the distribution of CITO test scores in 6th grade. All analyses are split by parental net worth at baseline. Panels on the left compare the distribution of test scores between children whose fathers became unemployed in the winter v. spring quarter of 6th grade. Panels on the right present point estimates and 95% confidence intervals for quantile treatment effects (Borgen et al., 2022) of paternal unemployment in the winter of 6th grade (ref. 5th grade). The outcome is smoothed (Machado & Silva, 2005) and standard errors are estimated via bootstrapping with 50 replications.

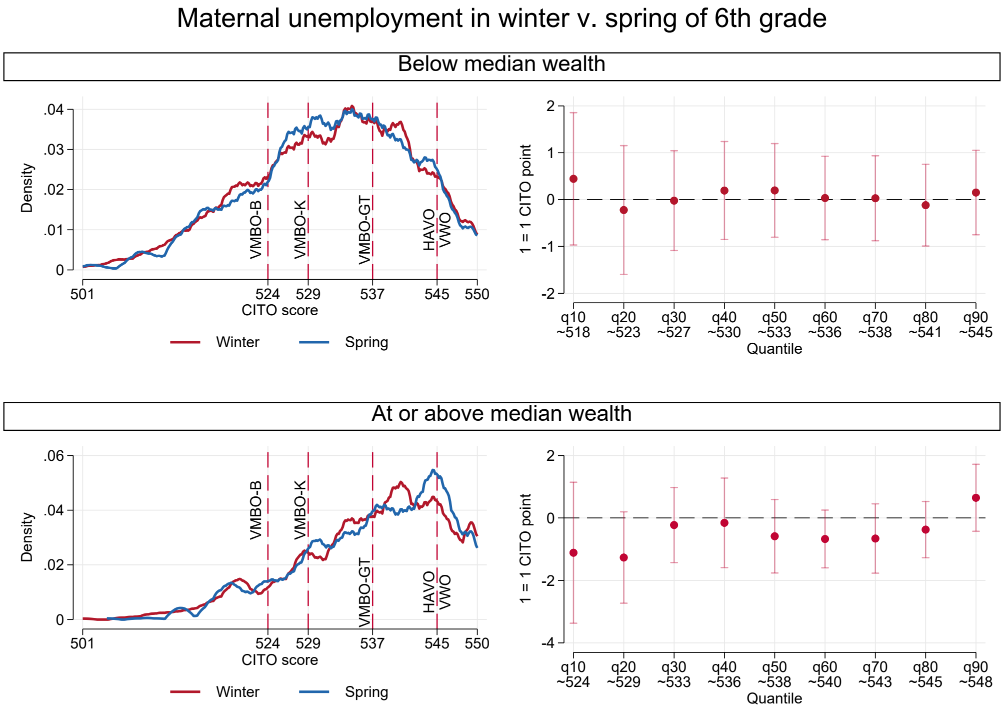

Figure 7A: Timing of maternal unemployment and the distribution of CITO test scores in 6th grade. All analyses are split by parental net worth at baseline. Panels on the left compare the distribution of test scores between children whose mothers became unemployed in the winter v. spring quarter of 6th grade. Panels on the right present point estimates and 95% confidence intervals for quantile treatment effects (Borgen et al., 2022) of maternal unemployment in the winter of 6th grade (ref. 5th grade). The outcome is smoothed (Machado & Silva, 2005) and standard errors are estimated via bootstrapping with 50 replications.

5. The timing of parental unemployment and enrolment in HAVO and VWO

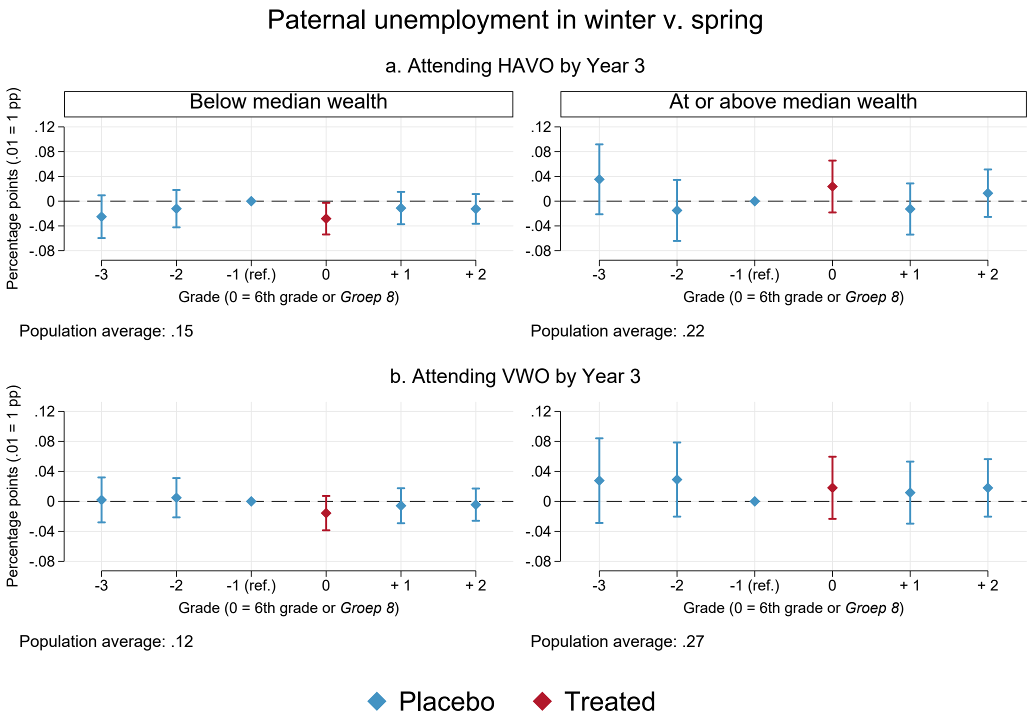

Figure 8A: Point estimates and 95% confidence intervals for the differential effects of the timing of paternal unemployment in 6th grade by unemployment benefit amounts. The outcome is the probability of attending HAVO (top) or VWO (bottom) by the third year of secondary school. Models are estimated as per Equation 1, plus an interaction with unemployment benefit amounts, separately by net worth at baseline and with robust standard errors.

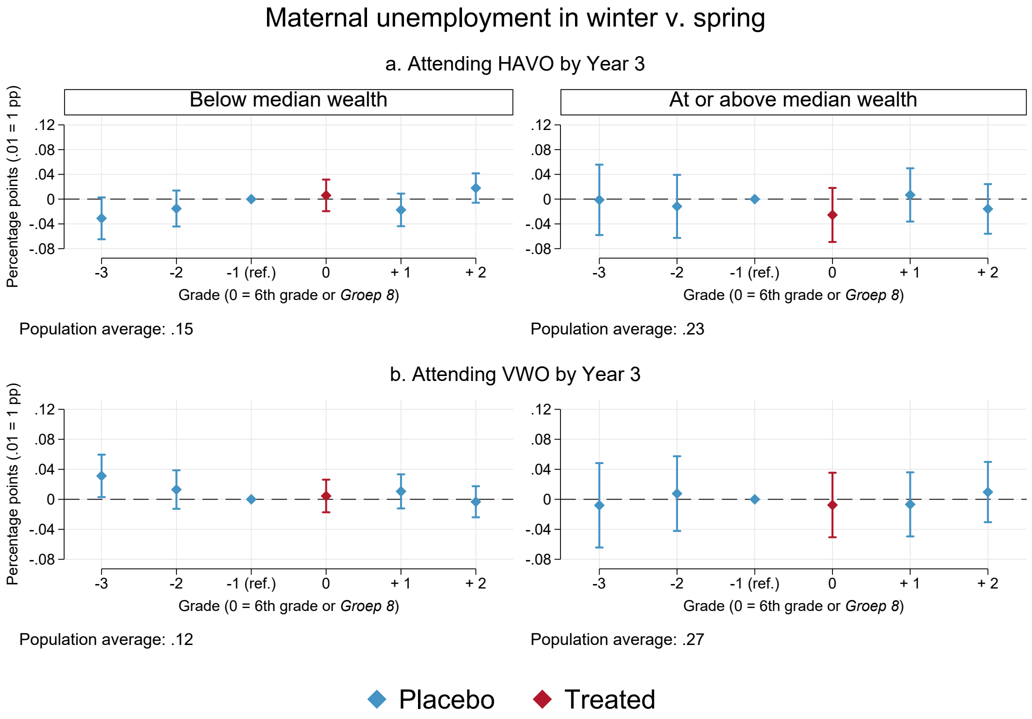

Figure 9A: Point estimates and 95% confidence intervals for the differential effects of the timing of maternal unemployment in 6th grade by unemployment benefit amounts. The outcome is the probability of attending HAVO (top) or VWO (bottom) by the third year of secondary school. Models are estimated as per Equation 1, plus an interaction with unemployment benefit amounts, separately by net worth at baseline and with robust standard errors.

6. Robustness and sensitivity checks

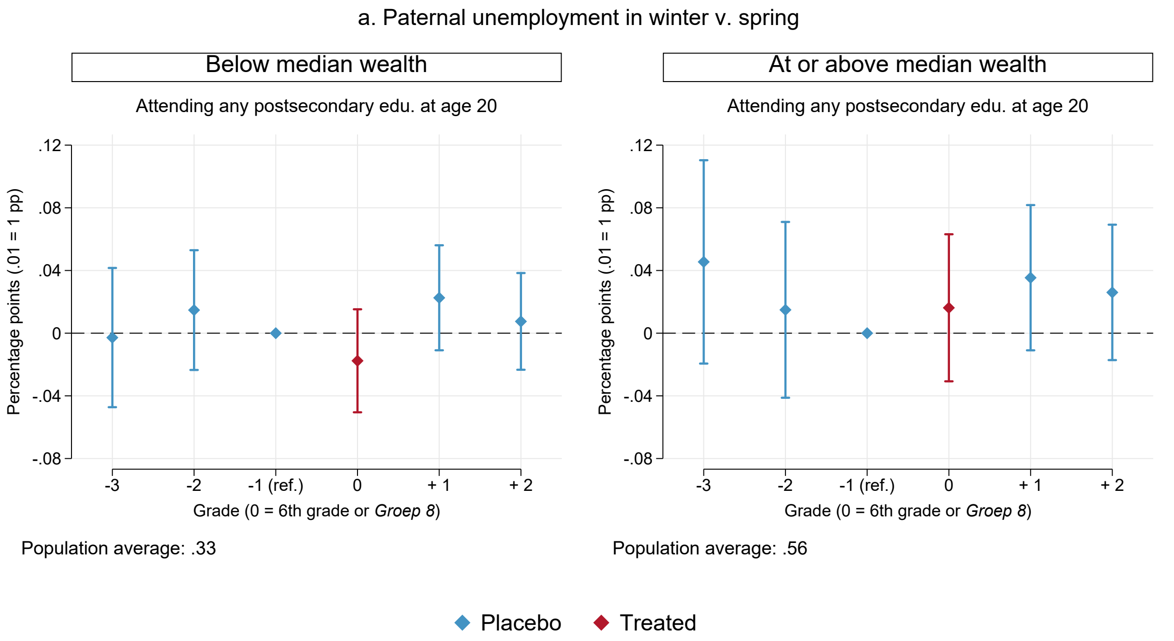

Figure 10A: Point estimates and 95% confidence intervals for the effects of the timing of paternal unemployment on the chances of attending any postsecondary education by age 20. Models are estimated as per Equation 1, with robust standard errors.

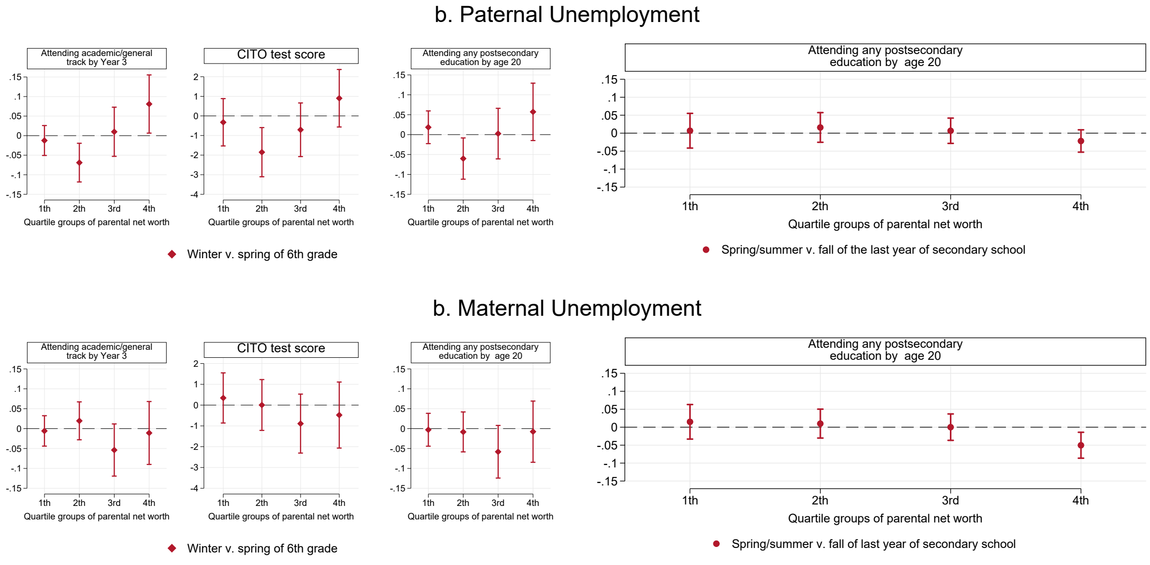

Figure 11A: Point estimates and 95% confidence intervals for the effects of the timing of parental unemployment on children's educational outcomes. Separate models by quartile group of parental net worth at baseline. Models are estimated as per Equation 1, with robust standard errors.

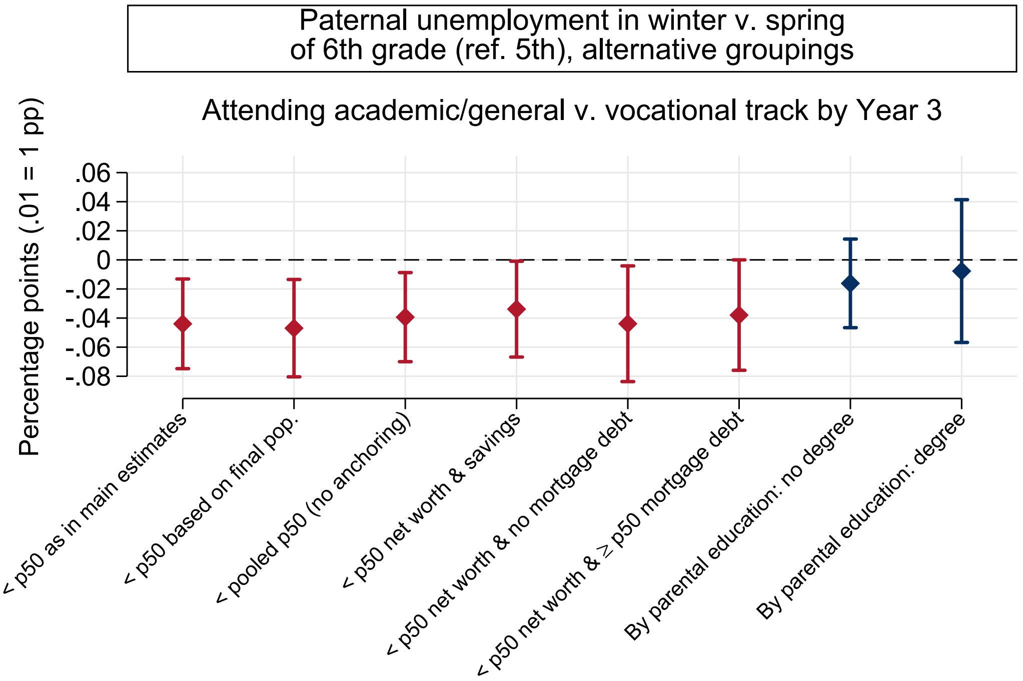

Figure 12A: Point estimates and 95% confidence intervals for the effects of the timing of paternal unemployment in 6th grade on children's chances of attending academic/general v. vocational track by the third year of secondary school. Models are ran separately for different sub-populations, as indicated on the x axis. "p50" refers to the median of the distribution of a given wealth measure. Models are estimated as per Equation 1 with robust standard errors.

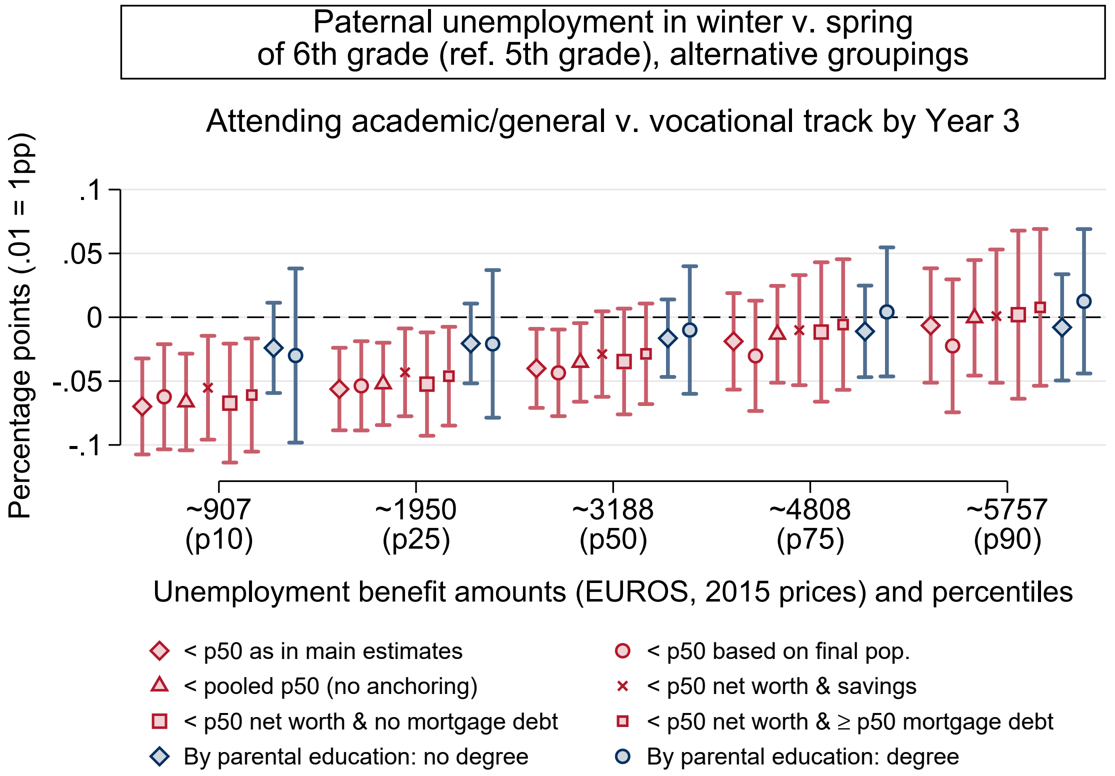

Figure 13A: Point estimates and 95% confidence intervals for the differential effects of the timing of parental unemployment in 6th grade by unemployment benefit amounts. The outcome is the probability of attending academic/general v. vocational track by the third year of secondary school. Models are ran separately for different sub-populations, as indicated in the legend. "p50" refers to the median of the distribution of a given wealth measure. Models are estimated as per Equation 1, plus an interaction with unemployment benefit amounts, separately by net worth at baseline and with robust standard errors.

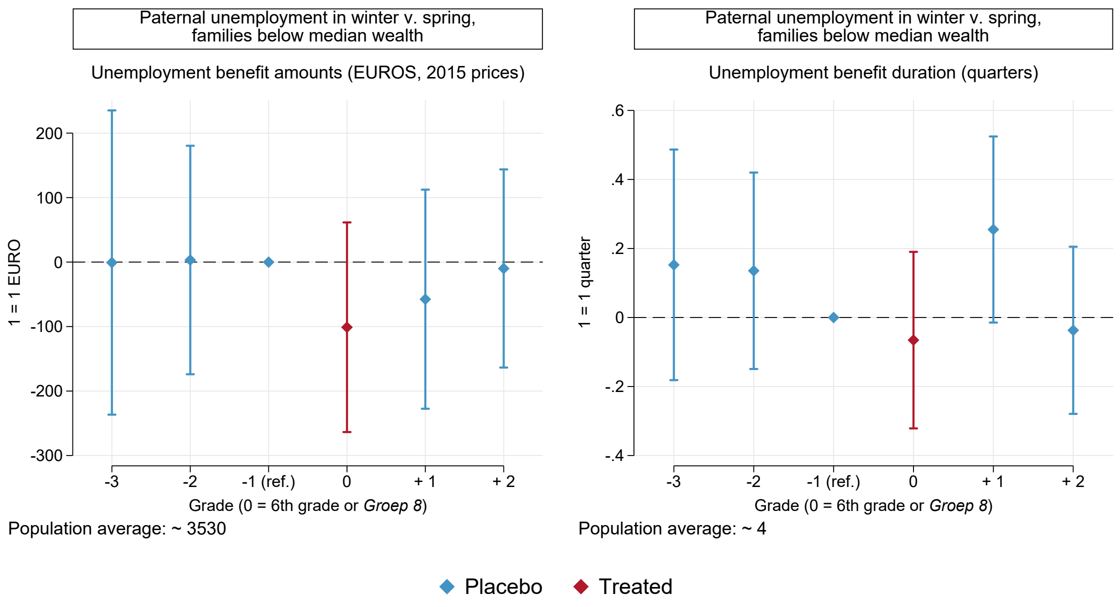

Figure 14A: Point estimates and 95% confidence intervals for the effects of the timing of paternal unemployment on unemployment benefit amounts (left) and duration (right). Models are estimated as per Equation 1 with robust standard errors.

7. Comparison with population not affected by parental unemployment

Table 2A: Descriptive statistics (means/proportions) for children born 1992-1998 who did not experience a spell of parental unemployment.

	At or above median wealth	Below median wealth	Total
Girl	.49	.50	.49
Born in the Netherlands	.99	.97	.98
Twin birth	.03	.03	.03
Birth order	1.4	1.3	1.4
Parental education: Degree	.75	.63	.71
Household type†: Cohabiting couple	.05	.08	.06
Household type†: Married couple	.93	.89	.92
Household type†: Single parent	.01	.03	.02
Parental wealth†: Below 2006 median			.36
Paternal employment, last 48 months†:			
Continuous (v. intermittent)	.95	.91	.94
Parent received†			
Social assistance	.0001	.02	.01
Sickness/disability benefit	.02	.03	.02
Other social security	.003	.01	.004
Attended general/academic track by Year 3	.52	.39	.47
N	395,785	224,963	620,748
Enrolled in postsecondary edu. at 20††	.89	.85	.88
N	206,596	86,707	293,303
CITO	537	534	536
N	225,170	125,390	350,560

† Baseline values refers to the child's father and the first quarter of 2006.

†† If attending HAVO/VWO in Year 3.

8. Main tables

Table 3A: Timing of parental unemployment and children's chances of being enrolled in general/academic tracks by the third year of secondary school. Linear models (Equation 1), robust standard errors in parentheses (see Figure 1 and 2 in the main text).

	Paternal unemployment			Maternal unemployment		
	All	Lower wealth	Higher wealth	All	Lower wealth	Higher wealth
- 3	0.006	−0.023	0.063	−0.003	0.000	−0.009
	(0.018)	(0.021)	(0.033)	(0.017)	(0.020)	(0.033)
- 2	−0.001	−0.007	0.014	−0.003	−0.002	−0.004
	(0.016)	(0.018)	(0.028)	(0.015)	(0.018)	(0.029)
- 1 (ref.)						
6th grade/*Groep8* (0)	−0.014	−0.044**	0.042	−0.003	0.011	−0.033
	(0.013)	(0.016)	(0.024)	(0.013)	(0.016)	(0.025)
+ 1	−0.013	−0.017	−0.001	−0.004	−0.007	0.000
	(0.013)	(0.016)	(0.024)	(0.014)	(0.016)	(0.025)
+ 2	−0.001	−0.017	0.031	0.008	0.015	−0.006
	(0.012)	(0.015)	(0.022)	(0.012)	(0.015)	(0.023)
Controls	✓	✓	✓	✓	✓	✓
Year and grade FE	✓	✓	✓	✓	✓	✓
Observations	44,637	28,023	16,614	43,851	28,666	15,185

* $p < .05$, ** $p < .01$, *** $p < .001$.

Table 4A: Timing of parental unemployment in 6th grade and children's chances of being enrolled in general/academic tracks by the third year of secondary school. Predicted effects by quantile of unemployment benefit amounts (ref. 5th grade). Linear models (Equation 1 plus an interaction between unemployment timing and benefit amounts), robust standard errors in parentheses (see Figure 3 in the main text).

	Paternal unemployment		Maternal unemployment	
	Lower wealth	Higher wealth	Lower wealth	Higher wealth
p10	-0.070^{***}	0.040	0.002	-0.016
	(0.019)	(0.031)	(0.019)	(0.031)
p25	-0.056^{***}	0.041	0.005	-0.021
	(0.016)	(0.027)	(0.017)	(0.028)
p50	-0.040^{*}	0.042	0.009	-0.030
	(0.016)	(0.024)	(0.016)	(0.026)
p75	-0.019	0.044	0.016	-0.041
	(0.019)	(0.025)	(0.017)	(0.026)
p90	-0.006	0.045	0.023	-0.053
	(0.023)	(0.027)	(0.022)	(0.032)
Controls	✓	✓	✓	✓
Year and grade FE	✓	✓	✓	✓
Observations	28,023	16,614	28,666	15,185

* $p < .05$, ** $p < .01$, *** $p < .001$.

Table 5A: Timing of parental unemployment and children's chances of being enrolled in post-secondary education at age 20. Linear models (Equation 1), robust standard errors in parentheses (see Figure 4 and 5 in the main text).

	Paternal unemployment			Maternal unemployment		
	All	Lower wealth	Higher wealth	All	Lower wealth	Higher wealth
- 3	0.008	0.012	0.006	−0.028*	−0.030	−0.028
	(0.012)	(0.019)	(0.015)	(0.013)	(0.019)	(0.016)
- 2	−0.021	−0.019	−0.023	−0.009	−0.001	−0.019
	(0.011)	(0.017)	(0.014)	(0.011)	(0.017)	(0.015)
- 1 (ref.)						
Last year of sec. school (0)	0.001	0.009	−0.005	−0.004	0.013	−0.027
	(0.010)	(0.015)	(0.013)	(0.010)	(0.015)	(0.014)
+ 1	−0.007	−0.006	−0.007	0.011	0.015	0.004
	(0.010)	(0.015)	(0.012)	(0.010)	(0.015)	(0.013)
+ 2	−0.003	0.008	−0.013	−0.003	0.007	−0.017
	(0.010)	(0.015)	(0.013)	(0.010)	(0.015)	(0.013)
Controls	✓	✓	✓	✓	✓	✓
Year and grade FE	✓	✓	✓	✓	✓	✓
Observations	38,328	19,083	19,245	38,216	20,392	17,824

* $p < .10$, ** $p < .05$, *** $p < .01$.

Table 6A: Timing of parental unemployment in 6th grade and children's chances of being enrolled in post-secondary education at age 20. Predicted effects by quantile of unemployment benefit amounts (ref. year prior to last). Linear models (Equation 1 plus an interaction between unemployment timing and benefit amounts), robust standard errors in parentheses (see Figure 6 in the main text).

	Paternal unemployment		Maternal unemployment	
	Lower wealth	Higher wealth	Lower wealth	Higher wealth
p10	0.018	−0.001	−0.008	−0.031
	(0.018)	(0.016)	(0.022)	(0.021)
p25	0.014	−0.003	−0.002	−0.032
	(0.016)	(0.014)	(0.021)	(0.019)
p50	0.010	−0.004	0.007	−0.032
	(0.015)	(0.013)	(0.019)	(0.017)
p75	0.005	−0.006	0.021	−0.034*
	(0.015)	(0.013)	(0.019)	(0.017)
p90	0.002	−0.007	0.040	−0.036
	(0.016)	(0.013)	(0.023)	(0.021)
Controls	✓	✓	✓	✓
Year and grade FE	✓	✓	✓	✓
Observations	19,083	19,245	12,567	10,753

* $p < .05$, ** $p < .01$, *** $p < .001$.
